# Amino acids serve as an important energy source for adult flukes of *Clonorchis sinensis*

**DOI:** 10.1371/journal.pntd.0008287

**Published:** 2020-04-30

**Authors:** Shan Li, Xueqing Chen, Juanjuan Zhou, Zhizhi Xie, Mei Shang, Lei He, Pei Liang, Tingjin Chen, Qiang Mao, Chi Liang, Xuerong Li, Yan Huang, Xinbing Yu

**Affiliations:** 1 Department of Pathology and Pathophysiology, Henan University of Chinese Medicine, Zhengzhou, Henan, People’s Republic of China; 2 Department of Parasitology, Zhongshan School of Medicine, Sun Yat-Sen University, Guangzhou, Guangdong, People’s Republic of China; 3 Key Laboratory for Tropical Diseases Control, Sun Yat-Sen University, Ministry of Education, Guangzhou, Guangdong, People’s Republic of China; 4 Clinical Laboratory, The First Affiliated Hospital of Guangzhou University of Chinese Medicine, Guangzhou, Guangdong, People’s Republic of China; 5 Zhengzhou Key Laboratory for Children’s Infection and Immunity, Children’s Hospital Affiliated to Zhengzhou University, Zhengzhou, Henan, People’s Republic of China; 6 Clinical Laboratory, Third Affiliated Hospital of Sun Yat-Sen University, Guangzhou, Guangdong, People’s Republic of China; Queen’s University Belfast, UNITED KINGDOM

## Abstract

Clonorchiasis, caused by chronic infection with *Clonorchis sinensis* (*C*. *sinensis*), is an important food-borne parasitic disease that seriously afflicts more than 35 million people globally, resulting in a socioeconomic burden in endemic regions. *C*. *sinensis* adults long-term inhabit the microaerobic and limited-glucose environment of the bile ducts. Energy metabolism plays a key role in facilitating the adaptation of adult flukes to crowded habitat and hostile environment. To understand energy source for adult flukes, we compared the component and content of free amino acids between *C*. *sinensis*-infected and uninfected bile. The results showed that the concentrations of free amino acids, including aspartic acid, serine, glycine, alanine, histidine, asparagine, threonine, lysine, hydroxylysine, and urea, were significantly higher in *C*. *sinensis*-infected bile than those in uninfected bile. Furthermore, exogenous amino acids could be utilized by adult flukes via the gluconeogenesis pathway regardless of the absence or presence of exogenous glucose, and the rate-limiting enzymes, such as *C*. *sinensis* glucose-6-phosphatase, fructose-1,6-bisphosphatase, phosphoenolpyruvate carboxykinase, and pyruvate carboxylase, exhibited high expression levels by quantitative real-time PCR analysis. Interestingly, no matter whether exogenous glucose was present, inhibition of gluconeogenesis reduced the glucose and glycogen levels as well as the viability and survival time of adult flukes. These results suggest that gluconeogenesis might play a vital role in energy metabolism of *C*. *sinensis* and exogenous amino acids probably serve as an important energy source that benefits the continued survival of adult flukes in the host. Our study will be a cornerstone for illuminating the biological characteristics of *C*. *sinensis* and the host-parasite interactions.

## Author summary

Clonorchiasis, closely related to cholangiocarcinoma and hepatocellular carcinoma, has led to a negative socioeconomic impact in global areas especially some Asian endemic regions. Owing to the emergence of drug resistance and hypersensitivity reactions after the massive and repeated use of praziquantel as well as the lack of effective vaccines, searching for new strategies that prevent and treat clonorchiasis has become an urgent matter. *Clonorchis sinensis*, the causative agent of clonorchiasis, long-term inhabits the microaerobic and limited-glucose environment of the bile ducts. Adequate nutrients are essential for adult flukes to resist the adverse condition and survive in the crowed habitat. Studies on energy metabolism of adult flukes are beneficial for further exploring host-parasite interactions and developing novel anti-parasitic drugs. Our results suggest that gluconeogenesis probably plays a vital role in energy metabolism of *Clonorchis sinensis* and exogenous amino acids might be an essential energy source for adult flukes to successfully survive in the host. Our foundational study opens a new avenue for explaining energy metabolism of *Clonorchis sinensis* and provides a valuable strategy that the gluconeogenesis pathway will be a potential and novel target for the prevention and treatment of clonorchiasis.

## Introduction

*Clonorchis sinensis* (*C*. *sinensis*) is an important food-borne parasite endemic in Southeast Asian countries, including China, Korea, Japan, and Vietnam. Clonorchiasis, caused by chronic infection with *C*. *sinensis*, has seriously afflicted more than 35 million people worldwide, including approximately 15 million people in China [1]. Most clonorchiasis cases occur through the ingestion of raw or undercooked freshwater fish harboring infective *C*. *sinensis* metacercariae. The metacercariae pass through the gastric environment and then excyst in the duodenum, and eventually the juvenile flukes migrate into the bile ducts, where they grow into adult flukes [2]. These long-lived flukes can provoke severe tissue damage and pathologic changes such as inflammation, epithelial hyperplasia, goblet cell metaplasia, periductal fibrosis, and biliary intraepithelial neoplasia [3]. More importantly, increasing epidemiological, histopathological, and experimental evidences have revealed that the morbidity of cholangiocarcinoma strongly correlates with the rate of *C*. *sinensis* infection [4–7]. Although the treatment of clonorchiasis is largely reliant on a safe and effective drug, praziquantel, the massive and repeated use of it in endemic areas, is leading to several emerging problems, such as drug resistance and hypersensitivity reactions [8–10]. Furthermore, community prevention of clonorchiasis is difficult to carry out because of the lack of an effective vaccine [11]. The continuing search for novel anthelmintics and prophylactics that target the vital function of *C*. *sinensis* has become an urgent matter. Since energy metabolism plays a key role in facilitating the adaptation of adult flukes to a hostile environment, a detailed knowledge of it is beneficial for us to develop novel anti-parasitic drugs. In the body of adult flukes, endogenous glucose is degraded through two major pathways, homolactic fermentation and carbon dioxide fixation, both of which can produce a large amount of ATP for the flukes to grow, reproduce, and inhabit the host [12, 13]. However, to date, it is unclear how adult flukes obtain an adequate energy source from the bile ducts, where they encounter a microaerobic and limited-glucose environment [14].

Gluconeogenesis is a metabolic process involving the de novo synthesis of glucose molecules from other carbon precursors during situations in which there is an enhanced oxidative utilization of glucose or in which there is a dietary insufficiency of carbohydrates to supply the body requirements for oxidative glucose metabolism [15]. It is well known that gluconeogenesis exists in mammals, certain plants, and microorganisms grown in the absence of glucose [16]. Previous studies have shown that all gene encoding enzymes involved in gluconeogenesis are present in the *C*. *sinensis* genome. Among these enzymes, *C*. *sinensis* glucose-6-phosphatase (*Cs*G-6-Pase), fructose-1,6-bisphosphatase (*Cs*FBPase), phosphoenolpyruvate carboxykinase (*Cs*PEPCK), and pyruvate carboxylase (*Cs*PC), all of which are key regulatory enzymes in gluconeogenesis, exhibit high expression levels at the adult fluke stage [14, 17]. Moreover, it was reported that *C*. *sinensis* adults could absorb exogenous glycine, proline, succinic acid, and acetate under glucose-free conditions [18–21], suggesting that adult flukes might be obliged to utilize exogenous non-carbohydrate moieties to satisfy energy requirements in the absence of glucose. Studies on the gluconeogenesis pathway of *C*. *sinensis* will help us to elucidate the energy source of adult flukes in the bile ducts.

In the present study, we compared the component and content of free amino acids between *C*. *sinensis*-infected and uninfected bile and further investigated the consumption of exogenous amino acids by *C*. *sinensis* adults in the absence or presence of glucose as well as the influence of gluconeogenesis inhibition on their viability and survival time. The collective information will be a cornerstone for illuminating the biological characteristics of *C*. *sinensis* and the host-parasite interactions.

## Materials and methods

### Ethics statement

Adult cats at 2–5 years old were purchased from an epidemic area of China (Guangzhou, Guangdong Province). Six-week-old male SD rats were purchased from the animal center of Sun Yat-Sen University. The rats were housed under a 12 h lighting cycle in a controlled environment (room temperature of 20–28°C, humidity of 40–70%). The animal care and use protocols were approved by the Animal Care and Use Committee of Sun Yat-Sen University (permit number SYXK (Guangdong) 2012–0081). All animal experiments were carried out in accordance with Guangdong Provincial Laboratory Animal Administration Measures and the Laboratory Animal Administration Regulations issued by Ministry of Science and Technology of the People’s Republic of China.

### Detection of free amino acids from bile samples

Cats were maintained a same diet for 3 days before the experiment to avoid the bile affected by feeding. The infection status of the cats was preliminarily assessed by analyzing the weight, the luster of the fur, the feces, and so on. Emaciated cats with matte fur and eggs found in the feces were assessed as *C*. *sinensis* infection. The liver and gallbladder were dissected from the cats and the flukes were collected to determine the infection [22]. Finally, 7 *C*. *sinensis*-infected cats were totally observed with 648 liver flukes, while 3 uninfected cats were not observed with liver flukes. Bile samples from *C*. *sinensis*-infected and uninfected cats were respectively collected. Each sample was mixed with 5% 5-sulfosalicylic acid (1:2 dilutions) and stored at 4°C overnight. After centrifugation for 20 min at 12,000 *g*, the protein precipitation was removed and the supernatant was purified by NOVA-PAK C18 column (Waters, USA) at 4°C. Each supernatant sample was subsequently mixed with a pH 2.2 citrate buffer (1:9 and 1:6 dilutions for infected and uninfected bile samples, respectively). Finally, free amino acids in 20 μl of each processed sample (C_p_) were detected by an automatic amino acid analyzer (Hitachi, Japan) according to the manufacturer’s protocol. Concentration of free amino acids in each bile sample (C_b_) was calculated using equation C_b_(mg/L) = C_p_ × dilution multiples. The experiment was performed according to JY/T019-1996 general principles for amino acid analysis at 23.5°C and humidity of 64%.

### In vitro maintenance of *C*. *sinensis* adults

*C*. *sinensis* metacercariae were collected from experimentally infected freshwater *Pseudorasbora parva* in our ecologic pool [23]. The fish was digested with artificial gastric juice (0.2% HCl, 0.6% pepsin, pH 2.0) at 37°C for 2 h followed by filtration through a sieve mesh. After washing several times with sterile phosphate-buffered saline (PBS), the living metacercariae were collected under a light microscope. Each SD rat was artificially infected with 50 metacercariae and sacrificed 8 weeks later. Adult flukes were freshly harvested from the bile ducts of rats and washed 5 times in sterile PBS containing 100 U/ml penicillin and 100 mg/ml streptomycin to remove host contaminants. Subsequently, a total of 630 adult flukes were randomly placed 5 per well with 2 ml of sterile culture solutions in 6-well plates, and cultured at 37°C with 5% CO_2_. The culture solutions for maintenance of adult flukes in vitro were inorganic 1 × Locke’s with or without the addition of different nutrients as well as Dulbecco’s modified Eagle’s Medium (DMEM; Invitrogen, USA) with different concentrations of glucose [24]. The high, low, and no glucose DMEM contained 25, 5.6, and 0 mM glucose, respectively. All culture solutions contained 100 U/ml penicillin and 100 mg/ml streptomycin and replaced in every 1 d interval. Each culture solution was performed in sextuplicate and repeated with 3 plates. Viability and percent survival of adult flukes were monitored daily as described previously [25]. Freshly harvested adult flukes (0 h/d) and adult flukes at 1, 3, 5 and 7 d of culture were washed > 3 times in sterile PBS to get rid of possible contamination. Adult flukes were immersed in Sample Protector (TaKaRa, Japan) for RNA extraction and stored at -80°C before use.

### Quantitative real time PCR (qRT-PCR) analysis of *Cs*G-6-Pase, *Cs*FBPase, *Cs*PEPCK, and *Cs*PC

Total RNA was extracted from adult flukes using TRIZOL reagent (Invitrogen, USA) according to the manufacturer’s protocol. The concentration of RNA was detected by a nucleic acid/protein analyzer (Beckman Coulter, USA). The first-strand cDNA was synthesized from 1 μg of total RNA using the reverse transcriptase superscript (Fermentas, USA). Real-time PCR was performed on a Bio-Rad iQ5 instrument using SYBR Premix Ex Taq Kit (TaKaRa, Japan) according to the manufacturer’s protocol. Primers of *Cs*G-6-Pase, *Cs*FBPase, *Cs*PEPCK, and *Cs*PC (GenBank accession numbers: DF144183.1, DF144433.1, DF142981.1, and DF144082.1) were designed using the Primer Premier 5.0 software (S1 Table). *C*. *sinensis* β-actin (GenBank accession number: EU109284.1) was used as the reference gene for relative quantification (S1 Table) [26]. The specificity of each primer pair was examined by generated melting curve upon the completion of each gene amplification. The amplification profile consisted of 95°C for 30 s, 40 cycles of 95°C for 5 s, and 60°C for 20 s. Each gene amplification was performed in triplicate and the experiment was repeated for 3 times. Data were calculated using a Bio-Rad iQ5 Optical System Software (version 2.1) according to the 2^-ΔΔCt^ method [27].

### Assay of L-amino acid level in cultured supernatant of different DMEM

At 0, 6, 12, and 24 h of feeding, the supernatant of DMEM was collected and centrifuged for 10 min at 12,000 g to remove debris. The processed supernatant samples were stored at -80°C before use. L-amino acid level was quantified with L-Amino Acid Quantitation Colorimetric/Fluorometric Kit (BioVision, USA) according to the manufacturer’s protocol. Briefly, L-amino acid standard was diluted to a series of gradients in a final volume of 50 μl with L-amino acid buffer in a 96-well microplate. Three μl processed samples were added into the microplate and adjusted to 50 μl with L-amino acid buffer. L-amino acid standard and samples were respectively incubated with 50 μl reaction mix for 30 min at 37°C, protect from light. The fluorescence was measured at Ex/Em = 535/590 nm in a microplate reader. L-amino acid standard curve was obtained based on the standard concentration and the standard reading. The blank reading was subtracted from the sample readings to calculate L-amino acid level of samples by using the standard curve. Every sample was performed in sextuplicate and the experiment was repeated for 3 times.

### Inhibition of recombinant *Cs*FBPase (r*Cs*FBPase) and native *Cs*FBPase activity by MB05032

MB05032 (CAS No. 261365-11-1), [5-[2-amino-5-(2-methylpropyl)-4-thiazolyl]-2-furanyl] phosphonic acid, a specific inhibitor for human FBPase [28], was synthesized at Shanghai Medicilon Inc (China). For inhibitory potency assay, r*Cs*FBPase was prepared and purified as described [29], and soluble protein of adult flukes (0 d) was obtained essentially as described except that the buffer was 100 mM Tris-HCL (pH 7.5) [30]. Briefly, adult flukes were prepared by sonication and centrifuged at 12,000 *g* for 20 min at 4°C to clear off the insoluble matter. The supernatant containing soluble protein was collected. Enzymatic activity of r*Cs*FBPase or native *Cs*FBPase in soluble protein of adult flukes was respectively measured by continuously monitoring the rate of NADP^+^ reduction at 340 nm in a coupled assay using a multifunctional microplate reader (SpectraMax M5) [31]. The reaction mixture contained 100 mM Tris-HCL (pH 7.5), 2 mM MgCl_2_, 10 mM KCl, 0.4 mM FBP, 0.5 mM NADP^+^, 10 units/ml yeast glucose-6-phosphate dehydrogenase, and 4 units/ml yeast phosphoglucose isomerase (Sigma-Aldrich) in a final volume of 150 μl. The 50% inhibiting concentration (IC50) of AMP (positive control) and MB05032 against r*Cs*FBPase or native *Cs*FBPase in soluble protein was respectively measured at 37°C in a reaction mixture containing different concentrations of AMP (0–50 mM) or MB05032 (0–500μM). For inhibitory specificity assay, recombinant fructose-1,6-bisphosphate aldolase-2 of *C*. *sinensis* (r*Cs*FbA-2, GenBank accession number: DF143896.1) was prepared and used as a control [32]. Reaction was performed at 37°C in the reaction mixture of r*Cs*FBPase or r*Cs*FbA-2 with or without the addition of 10 μM MB05032. The reaction mixture of r*Cs*FbA-2 was prepared as described [32]. Every sample was performed in triplicate and the experiment was repeated for 3 times.

### Treatment of *C*. *sinensis* adults with or without MB05032

A total of 720 adult flukes were cultured as described above on 1 × Locke’s as well as high, low, and no glucose DMEM with or without the addition of MB05032 (20 μM). Viability and percent survival of adult flukes with or without MB05032 treatment were measured as described [25]. The pictures and videos of adult flukes were captured at 5 d of feeding using a stereoscopic dissecting microscope (Leica S8APO, Germany) for the investigation of motility. At different times of culture, adult flukes from each group were collected, washed > 3 times in sterile PBS to get rid of possible contamination and stored at -80°C until further processing.

### Assay of native *Cs*FBPase activity as well as glucose and glycogen levels

At 12 and 24 h of culture, native *Cs*FBPase activity in soluble protein of adult flukes with or without MB05032 treatment was measured using the same method as described above. Glucose and glycogen levels in soluble protein of adult flukes were detected with QuantiChrom^™^ Glucose Assay Kit and EnzyChrom^™^ Glycogen Assay Kit (Bioassay, USA), respectively. For detection of glucose, glucose standard was diluted to a series of concentration gradients in a final volume of 150 μl with dH_2_O. Then, 50 μl standards and samples were respectively mixed with 500 μl reagent in appropriately labeled tubes. The tubes were heated in a boiling water bath for 8 min and cooled down in an ice water bath for 4 min. Subsequently, 200 μl processed standards and samples were respectively transferred into a 96-well microplate and read optical density at 620–650 nm. Standard curve was obtained based on the standard concentration and the standard reading. The blank reading was subtracted from the sample readings to calculate glucose level of samples by using the standard curve. For detection of glycogen, glycogen standard was diluted to a series of concentration gradients in a final volume of 200 μl with dH_2_O. Then, 10 μl standards and samples were respectively transferred into separate wells of a 96-well microplate, and incubated with 90 μl work reagent for 30 min at room temperature, protect from light. The fluorescence was measured at Ex/Em = 530/585 nm. Standard curve was obtained based on the standard concentration and the standard reading. The blank reading was subtracted from the sample readings to calculate glycogen level of samples by using the standard curve. Protein concentration was measured with BCA protein assay kit (Novagen, Germany) and used for the normalization of glucose and glycogen levels as described [33]. Every sample was performed in triplicate and the experiment was repeated for 3 times.

### Statistical analysis

All data were analyzed using SPSS 13.0 software and the graphs were constructed using GraphPad Prism 5.0 software. Data were represented as mean ± standard deviation (SD) from 3 independent experiments. *P* < 0.05 was considered as statistical significance, thereby indicating a probability of error lower than 5%.

## Results

### Comparison of free amino acids between *C*. *sinensis*-infected and uninfected bile

To understand energy source for *C*. *sinensis* adults, we compared the component and content of free amino acids between *C*. *sinensis*-infected and uninfected bile. It was apparent that total concentration of free amino acids in *C*. *sinensis*-infected bile was 0.8 folds higher than that in uninfected bile. In *C*. *sinensis*-infected bile, the concentrations of aspartic acid, serine, glycine, alanine, histidine, asparagine (Asp, Ser, Gly, Ala, His, and Asn; glycogenic amino acid), threonine (Thr; glycogenic and ketogenic amino acid), lysine (Lys; ketogenic amino acid), hydroxylysine (Hylys), and urea increased to approximately 2.2, 2.5, 1.9, 2.5, 4.4, 2.6, 2.2, 8.9, 4.4, and 2.1 folds, respectively. Additionally, carnosine could be detected in *C*. *sinensis*-infected bile, while it could not be detected in uninfected bile (Table 1 and S1 Fig).

**Table 1:** Comparison of free amino acids between *C*. *sinensis*-infected and uninfected bile.

Free amino acid	Uninfected bile (mg/L, n = 3)	*C*. *sinensis*-infected bile (mg/L, n = 7)	*P* value
**Aspartic acid (Asp)**^a^	7.85 ± 1.47	17.58 ± 2.44	< 0.05 (0.020)
**Serine (Ser)**^a^	49.23 ± 6.88	123.05 ± 30.63	< 0.05 (0.039)
**Glycine (Gly)**^a^	101.05 ± 5.40	187.37 ± 20.82	< 0.05 (0.017)
**Alanine (Ala)**^a^	91.00 ± 16.82	230.68 ± 49.12	< 0.05 (0.039)
**Histidine (His)**^a^	6.14 ± 1.62	27.22 ± 7.83	< 0.05 (0.030)
**Asparagine (Asn)**^a^	29.07 ± 3.66	74.59 ± 15.91	< 0.05 (0.018)
**Valine (Val)**^a^	27.19 ± 8.59	52.64 ± 12.80	n.s. (0.305)
**Cysteine (Cys)**^a^	8.89 ± 2.89	36.88 ± 15.27	n.s. (0.732)
**Methionine (Met)**^a^	4.22 ± 1.18	11.35 ± 4.09	n.s. (0.733)
**Glutamic acid (Glu)**^a^	43.45 ± 20.97	60.60 ± 16.66	n.s. (0.569)
**Threonine (Thr)**^b^	64.14 ± 12.91	141.96 ± 21.25	< 0.05 (0.039)
**Isoleucine (Ile)**^b^	4.93 ± 1.08	14.87 ± 6.38	n.s. (0.732)
**Tyrosine (Tyr)**^b^	1.98 ± 1.27	22.73 ± 10.21	n.s. (0.299)
**Phenylalanine (Phe)**^b^	1.59 ± 1.03	16.31 ± 7.56	n.s. (0.481)
**Lysine (Lys)**^c^	8.11 ± 4.86	72.33 ± 30.22	< 0.05 (0.039)
**Leucine (Leu)**^c^	9.52 ± 2.98	32.64 ± 13.33	n.s. (0.909)
**Ornithine (Orn)**	3.83 ± 2.73	13.96 ± 3.27	n.s. (0.087)
**Hydroxylysine (Hylys)**	0.85 ± 0.46	3.73 ± 0.46	< 0.05 (0.039)
**Phosphoserine (P-Ser)**	13.76 ± 6.34	27.48 ± 5.70	n.s. (0.305)
**Taurine (Tau)**	291.79 ± 122.58	301.97 ± 72.54	n.s. (0.606)
**Cystine (Cys-Cys)**	2.29 ± 1.65	4.27 ± 0.93	n.s. (0.360)
**Citrulline (Cit)**	3.72 ± 0.55	5.09 ± 2.20	n.s. (0.908)
**β-Alanine (β-Ala)**	5.62 ± 1.84	6.90 ± 0.55	n.s. (0.425)
**α-Aminoadipic acid (α-AAA)**	4.62 ± 0.89	6.43 ± 0.68	n.s. (0.087)
**α-Aminobutyric acid (α-ABA)**	8.83 ± 4.99	22.36 ± 7.06	n.s. (0.138)
**Ethanolamine (ETA)**	10.29 ± 4.60	15.29 ± 2.05	n.s. (0.210)
**Phosphorylethanolamine (PEA)**	12.61 ± 7.12	9.66 ± 4.65	n.s. (0.729)
**Urea**	218.66 ± 110.50	450.75 ± 33.76	< 0.05 (0.039)
**Carnosine**	-	19.84 ± 7.68	< 0.05 (0.034)
**Total**	1125.12 ± 193.20	2036.29 ± 176.01	< 0.05 (0.017)

Data were represented as mean ± SD. Two independent samples test was used to evaluate the comparisons. *P* < 0.05 was considered as statistical significance; n.s., not significant.

^a^Glycogenic amino acid.

^b^Glycogenic and ketogenic amino acid.

^c^Ketogetic amino acid.

### Survival time of *C*. *sinensis* adults maintained on 1 × Locke’s with or without the addition of different nutrients

**Fig 1:**
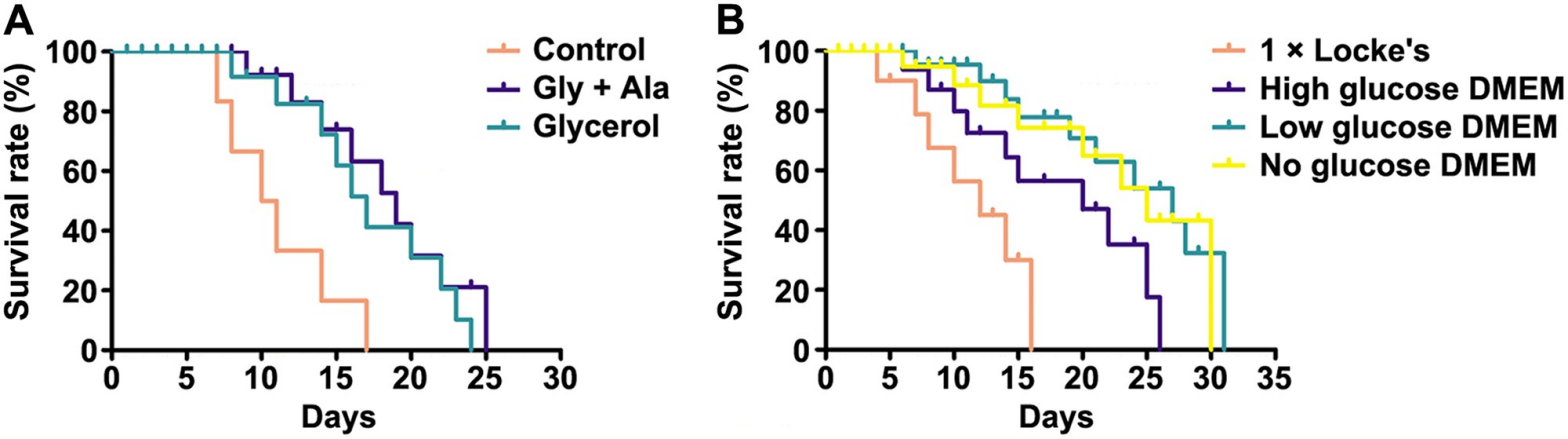
Survival curve of *C*. *sinensis* adults maintained on inorganic solutions and nutrient media. (A) Survival curve of adult flukes maintained on 1 × Locke’s (control), 1 × Locke’s containing 0.25% Gly and 0.25% Ala (Gly + Ala), as well as 1 × Locke’s containing 0.5% glycerol (glycerol). (B) Survival curve of adult flukes maintained on 1 × Locke’s and different DMEM. Survival curve was constructed using Kaplan-Meier analysis with the log-rank test.

To investigate whether exogenous glycogenic precursors could be utilized by adult flukes in the absence of glucose, we maintained adult flukes on inorganic 1 × Locke’s with or without the addition of 0.25% Gly and 0.25% Ala (Gly + Ala) or 0.5% glycerol (glycerol) [24]. Adult flukes maintained on 1 × Locke’s (control) did not survive beyond 18 d. Nevertheless, Gly + Ala feeding significantly extended the survival time of adult flukes (Gly + Ala versus control, *P* < 0.01), with median survival time increasing from 10.5 ± 0.5 to 19.1 ± 1.7 d and maximum survival time increasing from 17.8 ± 0.8 to 25.3 ± 1.5 d. Similarly, glycerol feeding significantly extended the survival time of adult flukes (glycerol versus control, *P* < 0.05), with median survival time increasing from 10.5 ± 0.5 to 17.0 ± 1.5 d and maximum survival time increasing from 17.8 ± 0.8 to 24.2 ± 1.0 d. Gly + Ala feeding respectively extended median and maximum survival times by approximately 81.9% and 42.1%, whereas glycerol feeding respectively extended median and maximum survival times by approximately 61.9% and 36.0%. However, no significant difference was observed between Gly + Ala and glycerol feeding (*P* = 0.4161) (Fig 1A and S2 Table).

### The mRNA levels of *Cs*G-6-Pase, *Cs*FBPase, *Cs*PEPCK, and *Cs*PC in 1 × Locke’s with or without the addition of different nutrients

**Fig 2:**
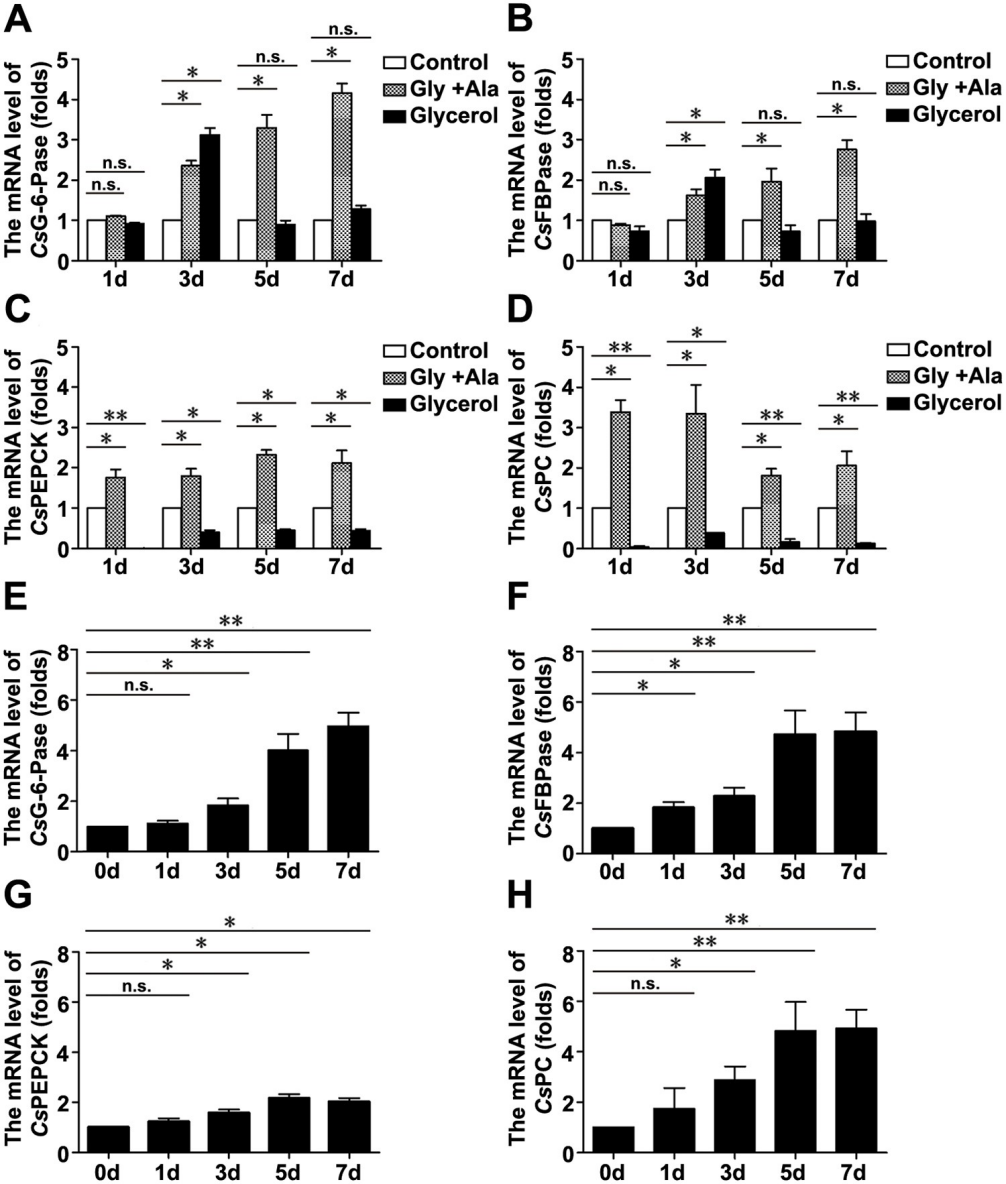
The mRNA levels of *Cs*G-6-Pase, *Cs*FBPase, *Cs*PEPCK, and *Cs*PC in 1 × Locke’s with or without the addition of different nutrients. The mRNA levels of *Cs*G-6-Pase (A), *Cs*FBPase (B), *Cs*PEPCK (C), and *Cs*PC (D). Time course of *Cs*G-6-Pase (E), *Cs*FBPase (F), *Cs*PEPCK (G), and *Cs*PC (H) mRNA levels in Gly + Ala. ANOVA was used to analyze the comparisons. *P < 0.05 and **P < 0.01 versus control (A-D) or 0 d (E-H); n.s., not significant.

Given the importance of *Cs*G-6-Pase, *Cs*FBPase, *Cs*PEPCK, and *Cs*PC in regulating the gluconeogenesis reaction, we analyzed their expression levels by qRT-PCR. Compared with the control, Gly + Ala feeding significantly upregulated the mRNA levels of *Cs*PEPCK and *Cs*PC at 1, 3, 5, and 7 d, and upregulated the mRNA levels of *Cs*G-6-Pase and *Cs*FBPase at 3, 5, and 7 d. However, glycerol feeding downregulated the mRNA levels of *Cs*PEPCK and *Cs*PC at 1, 3, 5, and 7 d, and upregulated the mRNA levels of *Cs*G-6-Pase and *Cs*FBPase only at 3 d (Fig 2A–2D). In addition, time course of *Cs*G-6-Pase, *Cs*FBPase, *Cs*PEPCK, and *Cs*PC mRNA levels in Gly + Ala was analyzed. Under Gly + Ala condition, the mRNA levels of *Cs*G-6-Pase, *Cs*FBPase, *Cs*PEPCK, and *Cs*PC increased progressively during 0–7 d of culture (Fig 2E–2H).

### Survival time of *C*. *sinensis* adults maintained on 1 × Locke’s and different DMEM

To further investigate whether exogenous amino acids could be utilized by adult flukes in the presence of glucose, we maintained adult flukes on 1 × Locke’s and DMEM with different concentrations of glucose. Adult flukes in any kind of DMEM had a longer survival time than adult flukes in 1 × Locke’s did (high glucose DMEM versus 1 × Locke’s, *P* < 0.05; low glucose DMEM versus 1 × Locke’s, *P* < 0.01; no glucose DMEM versus 1 × Locke’s, *P* < 0.01). Median and maximum survival times of adult flukes were 20.0 ± 1.0 and 25.8 ± 1.5 d in high glucose DMEM, 27.1 ± 1.5 and 32.2 ± 2.5 d in low glucose DMEM, and 24.9 ± 1.1 and 30.0 ± 2.0 d in no glucose DMEM, respectively, all of which were longer than those in 1 × Locke’s (median survival time, 12.0 ± 0.3 d; maximum survival time, 17.9 ± 1.2 d). Accordingly, median and maximum survival times were approximately prolonged by 66.7% and 44.1% in high glucose DMEM, 125.8% and 79.9% in low glucose DMEM, as well as 107.5% and 67.6% in no glucose DMEM, respectively. Moreover, adult flukes in high glucose DMEM survived shorter than those in low glucose DMEM survived (*P* < 0.05). However, no significant differences were observed between high and no glucose DMEM (*P* = 0.0974) as well as between low and no glucose DMEM (*P* = 0.6954) (Fig 1B and S3 Table).

### The mRNA levels of *Cs*G-6-Pase, *Cs*FBPase, *Cs*PEPCK, and *Cs*PC in 1 × Locke’s and different DMEM

**Fig 3:**
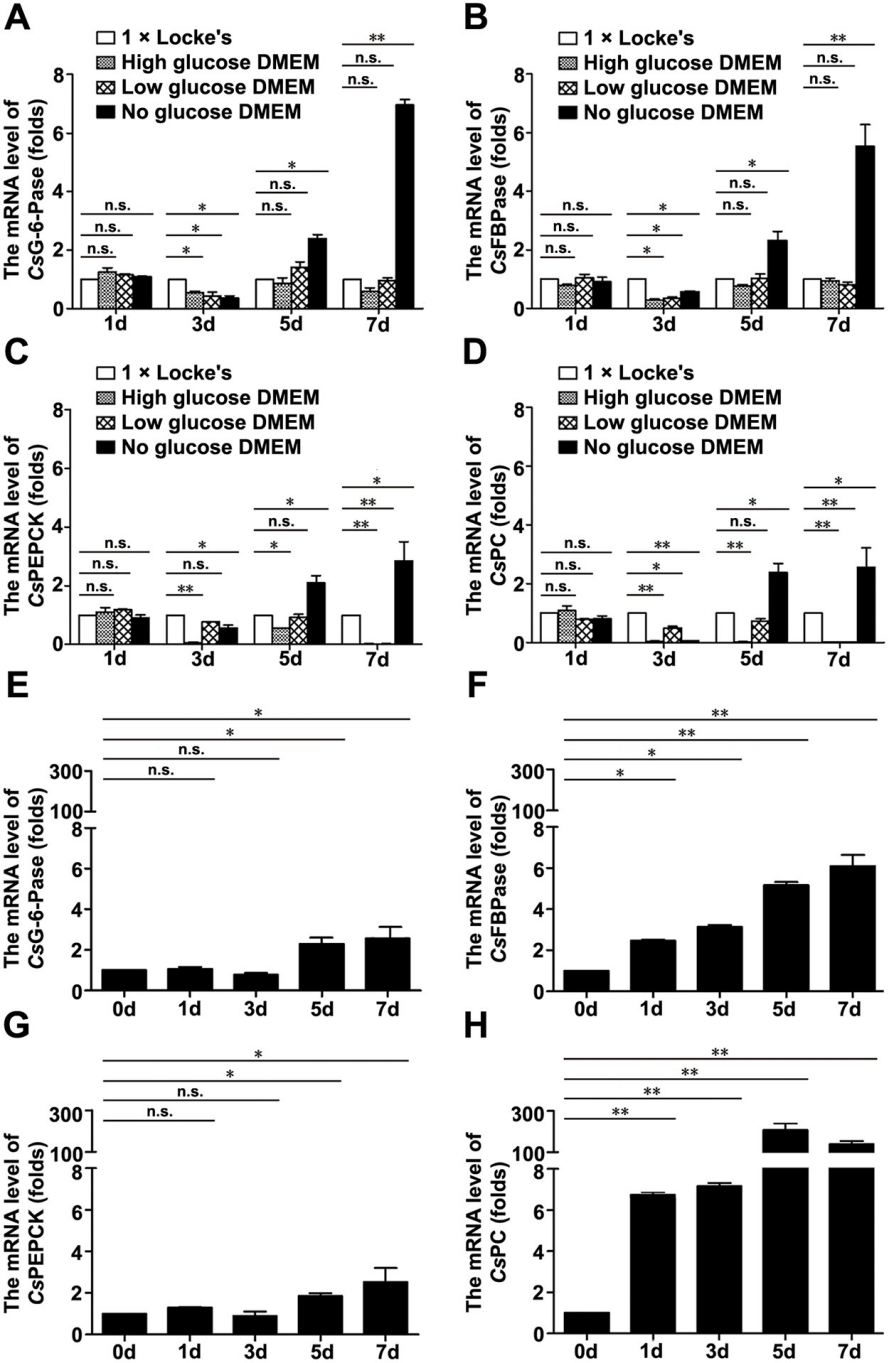
The mRNA levels of *Cs*G-6-Pase, *Cs*FBPase, *Cs*PEPCK, and *Cs*PC in 1 × Locke’s and different DMEM. The mRNA levels of *Cs*G-6-Pase (A), *Cs*FBPase (B), *Cs*PEPCK (C), and *Cs*PC (D). Time course of *Cs*G-6-Pase (E), *Cs*FBPase (F), *Cs*PEPCK (G), and *Cs*PC (H) mRNA levels in no glucose DMEM. ANOVA was used to analyze the comparisons. *P < 0.05 and **P < 0.01 versus 1 × Locke’s (A-D) or 0 d (E-H); n.s., not significant.

We analyzed the expression levels of *Cs*G-6-Pase, *Cs*FBPase, *Cs*PEPCK, and *Cs*PC in 1 × Locke’s and different DMEM by qRT-PCR to further determine the occurrence of gluconeogenesis inside the body of adult flukes. At 1, 3, 5, and 7 d, mRNA levels of *Cs*G-6-Pase, *Cs*FBPase, *Cs*PEPCK, and *Cs*PC were essentially consistent. At 1 d, mRNA levels of *Cs*G-6-Pase, *Cs*FBPase, *Cs*PEPCK, and *Cs*PC in high, low, and no glucose DMEM were not significantly changed as compared with those in 1 × Locke’s. At 3 d, adult flukes expressed the highest mRNA levels of *Cs*G-6-Pase, *Cs*FBPase, *Cs*PEPCK, and *Cs*PC in 1 × Locke’s. At 5 and 7 d, adult flukes expressed the highest mRNA levels of *Cs*G-6-Pase, *Cs*FBPase, *Cs*PEPCK, and *Cs*PC in no glucose DMEM. However, adult flukes did not express higher mRNA levels of *Cs*G-6-Pase, *Cs*FBPase, *Cs*PEPCK, and *Cs*PC in high and low glucose DMEM than those expressed in 1 × Locke’s (Fig 3A–3D). In addition, time course of *Cs*G-6-Pase, *Cs*FBPase, *Cs*PEPCK, and *Cs*PC mRNA levels in no glucose DMEM was analyzed. Under no glucose DMEM condition, the mRNA levels of *Cs*G-6-Pase, *Cs*FBPase, *Cs*PEPCK, and *Cs*PC increased progressively during 0–7 d of culture (Fig 3E–3H).

### L-amino acid level in cultured supernatant of different DMEM

**Fig 4:**
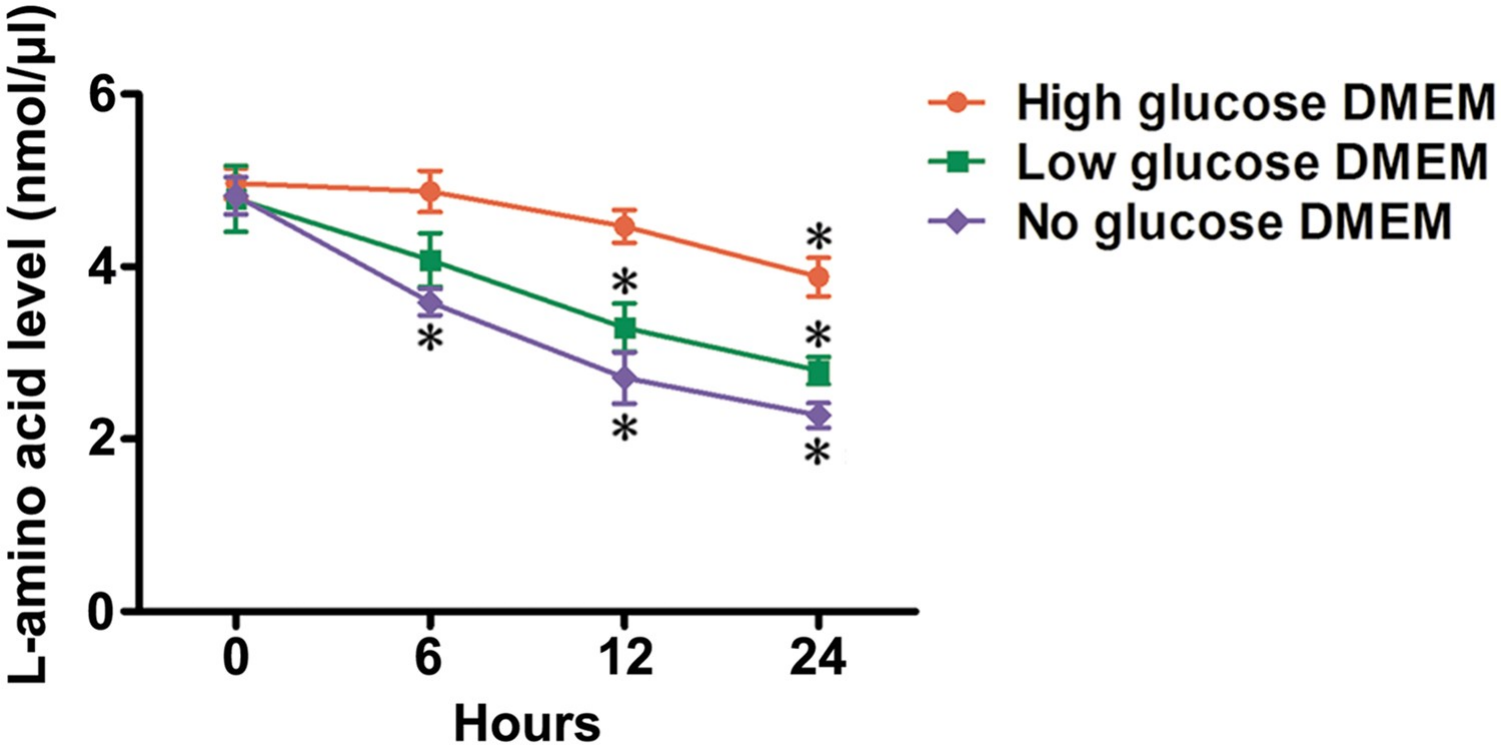
L-amino acid level in cultured supernatant of different DMEM. L-amino acid level in cultured supernatant of high, low, and no glucose DMEM was quantified at 0, 6, 12, and 24 h of feeding. Data were represented as means ± SD. Statistical significance was evaluated by ANOVA. *P < 0.05 versus 0 h.

To determine whether exogenous amino acids could be absorbed by adult flukes in the absence or presence of glucose, we detected L-amino acid level in cultured supernatant of different DMEM. L-amino acid level was gradually reduced in cultured supernatants of high, low, and no glucose DMEM during 0–24 h of feeding. Among these supernatants, the supernatant of no glucose DMEM showed a maximum reduction in L-amino acid level, while the supernatant of high glucose DMEM showed a minimum reduction in L-amino acid level (Fig 4).

### Specific inhibition of r*Cs*FBPase and native *Cs*FBPase by MB05032

The IC50 of AMP and MB05032 against r*Cs*FBPase were 262.2 μM and 4.898 μM, respectively (S2A Fig). The IC50 of AMP and MB05032 against native *Cs*FBPase were 116.7 μM and 2.531 μM, respectively (S2B Fig). In addition, r*Cs*FBPase activity could be inhibited by 10 μM MB05032. Conversely, r*Cs*FbA-2 activity could not be inhibited by 10 μM MB05032 (S2C Fig). These results indicated that r*Cs*FBPase activity could be efficaciously and specifically inhibited by MB05032.

### Native *Cs*FBPase activity inside *C*. *sinensis* adults with or without MB05032 treatment

**Fig 5:**
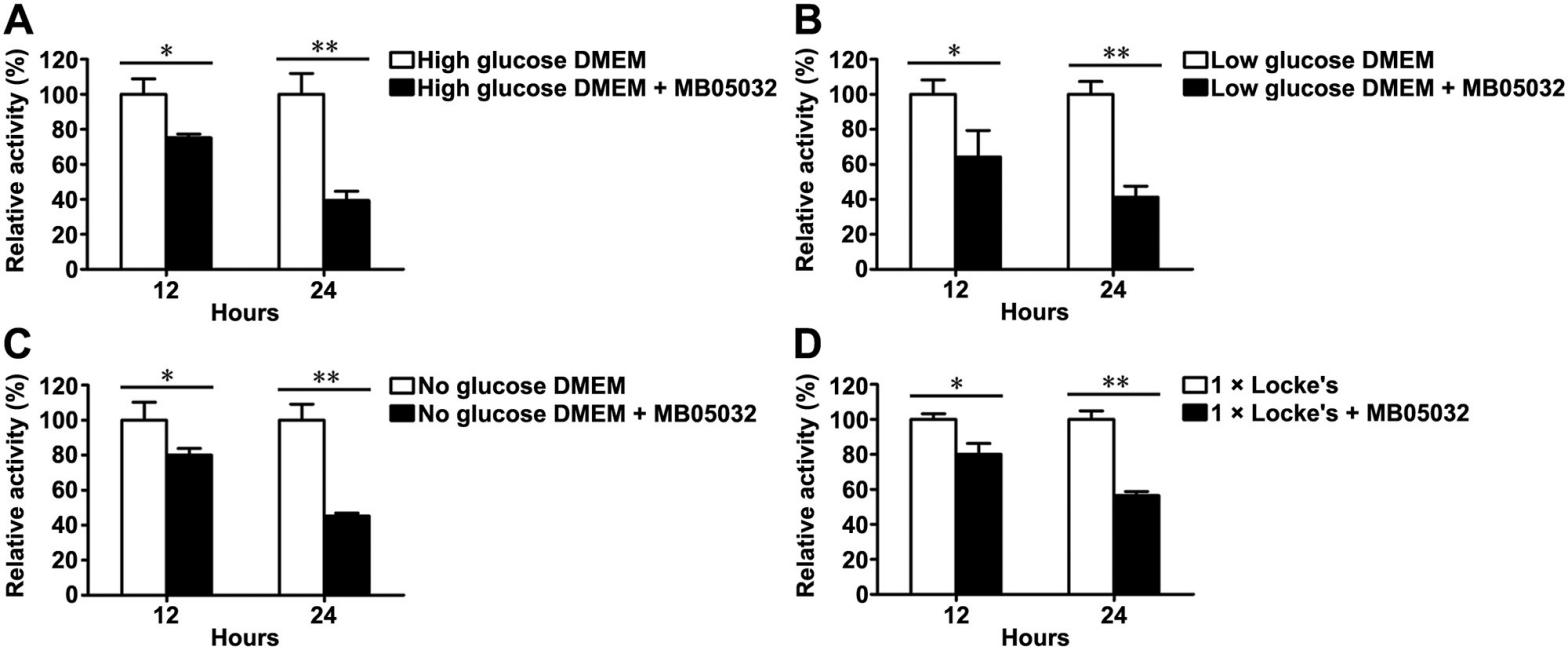
Native *Cs*FBPase activity inside *C*. *sinensis* adults with or without MB05032 treatment. Adult flukes were respectively maintained on high, low, and no glucose DMEM (A, B, and C), as well as 1 × Locke’s (D) with or without the addition of MB05032. Native *Cs*FBPase activity was measured at 12 and 24 h of feeding. Differences between two groups were evaluated by student’s *t* test. *P < 0.05 and **P < 0.01 were represented as statistical significance.

To further investigate the contribution of gluconeogenesis to energy metabolism of adult flukes, we maintained adult flukes on 1 × Locke’s and different DMEM with or without the addition of MB05032. Adult flukes treated with MB05032 exhibited a significant reduction in native *Cs*FBPase activity regardless of what kinds of culture solutions they were maintained (*P* < 0.05 or *P* < 0.01), with the relative activity reducing to less than 85% at 12 h and less than 60% at 24 h (Fig 5).

### Glucose and glycogen levels inside *C*. *sinensis* adults with or without MB05032 treatment

**Fig 6:**
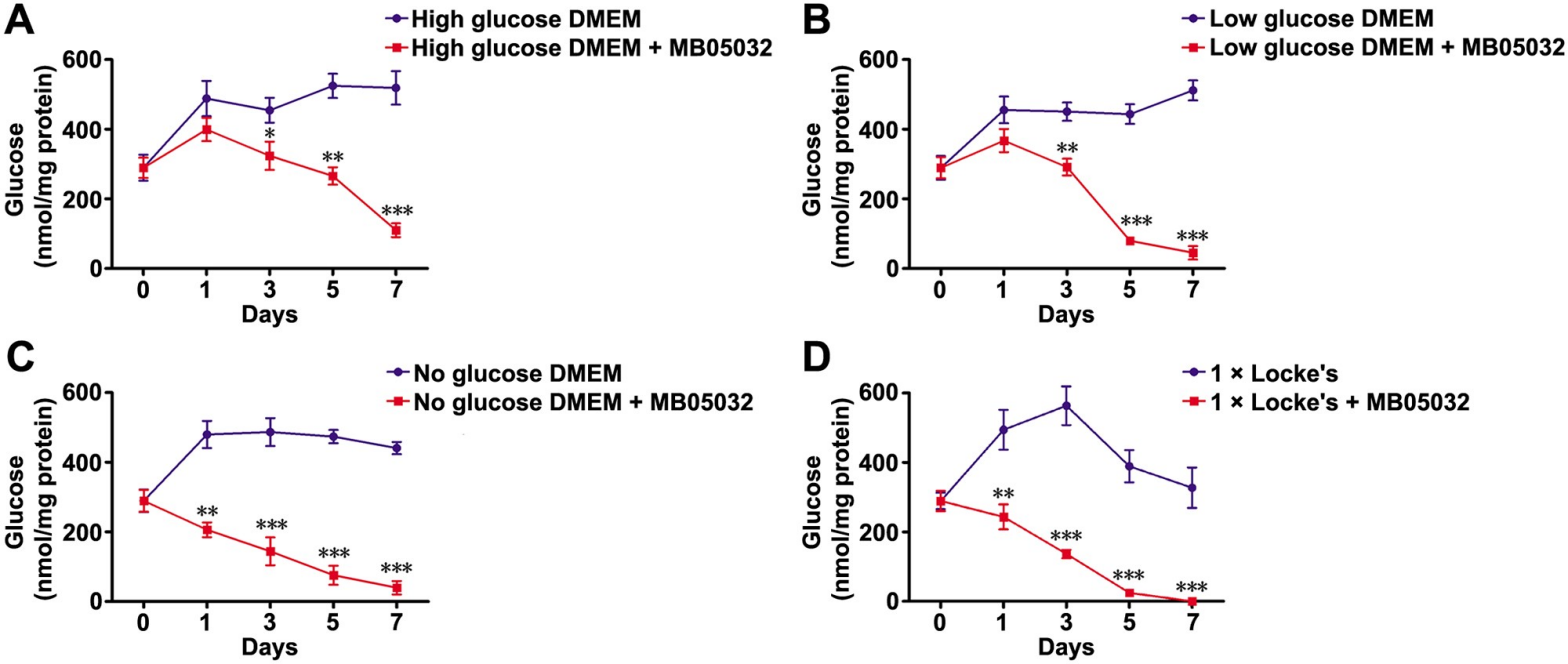
Glucose level inside *C*. *sinensis* adults with or without MB05032 treatment. Adult flukes were respectively maintained on high, low, and no glucose DMEM (A, B, and C), as well as 1 × Locke’s (D) with or without the addition of MB05032. Glucose level was detected at 0, 1, 3, 5, and 7 d of feeding. Differences between two groups were evaluated by student’s *t* test. *P < 0.05, **P < 0.01, and ***P < 0.001 were represented as statistical significance.

**Fig 7:**
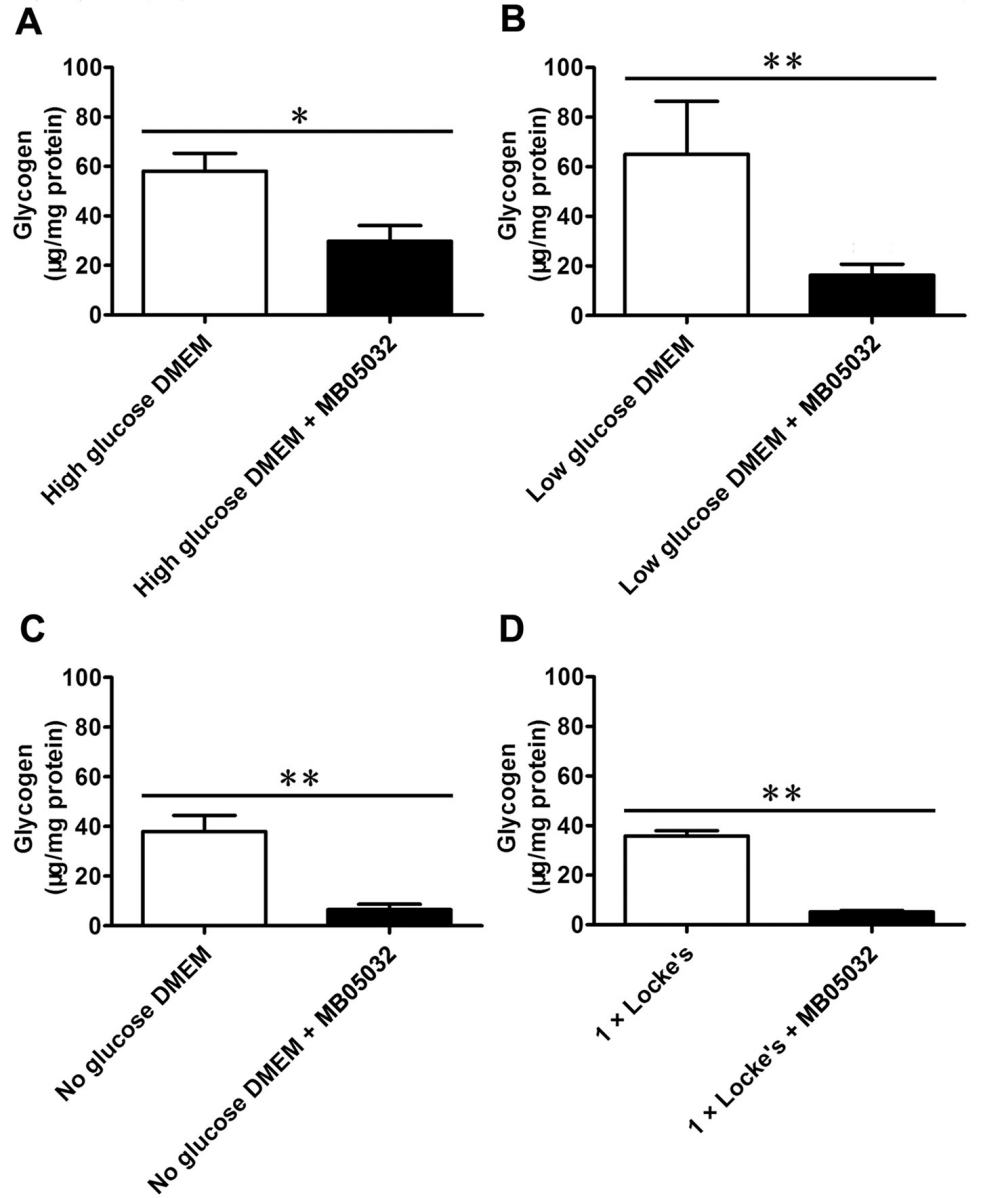
Glycogen level inside *C*. *sinensis* adults with or without MB05032 treatment. Adult flukes were respectively maintained on high, low, and no glucose DMEM (A, B, and C), as well as 1 × Locke’s (D) with or without the addition of MB05032. Glycogen level was detected at 24 h of feeding. Differences between two groups were evaluated by student’s *t* test. *P < 0.05 and **P < 0.01 were represented as statistical significance.

No matter what kind of culture solutions were used, MB05032 treatment decreased glucose level inside adult flukes, with more pronounced effect at 3, 5 and 7 d (*P* < 0.05, *P* < 0.01, or *P* < 0.001) (Fig 6). In addition to decreasing glucose level, MB05032 treatment also apparently decreased glycogen level inside adult flukes at 24 h of feeding, no matter what kind of culture solutions were used (*P* < 0.05 or *P* < 0.01) (Fig 7).

### Morphological and moving changes of *C*. *sinensis* adults with or without MB05032 treatment

**Fig 8:**
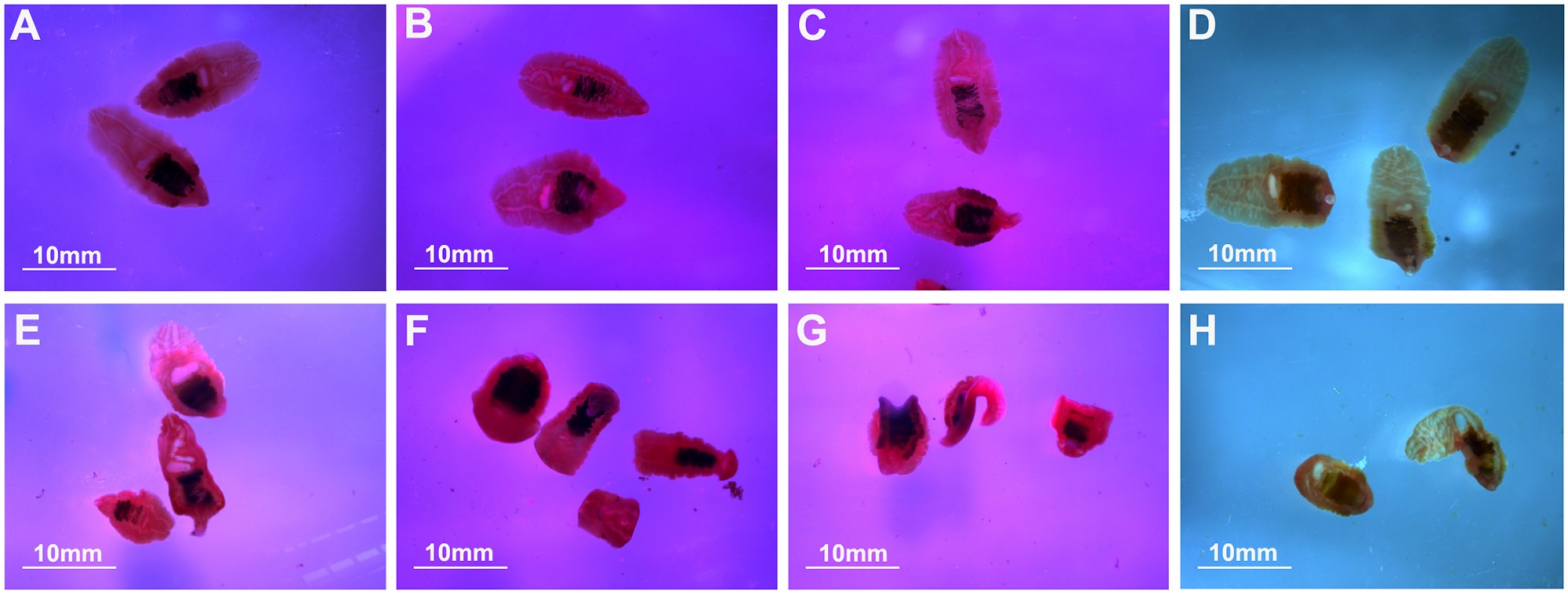
Morphological and moving changes of *C*. *sinensis* adults with or without MB05032 treatment. Adult flukes were maintained on high glucose DMEM (A), high glucose DMEM + MB05032 (E), low glucose DMEM (B), low glucose DMEM + MB05032 (F), no glucose DMEM (C), no glucose DMEM + MB05032 (G), 1 × Locke’s (D), and 1 × Locke’s + MB05032 (H), respectively. The images were captured at 5 d of feeding and magnified at × 32.

At 5 d, untreated adult flukes were active with continuous muscle contraction and expansion as well as body wriggle and migration. In contrast to this, adult flukes with MB05032 treatment showed reduced activities with body stiffness, shrink, and curliness regardless of what kind of culture solutions they were maintained (Fig 8 and S1–S8 Videos).

### Survival time of *C*. *sinensis* adults with or without MB05032 treatment

**Fig 9:**
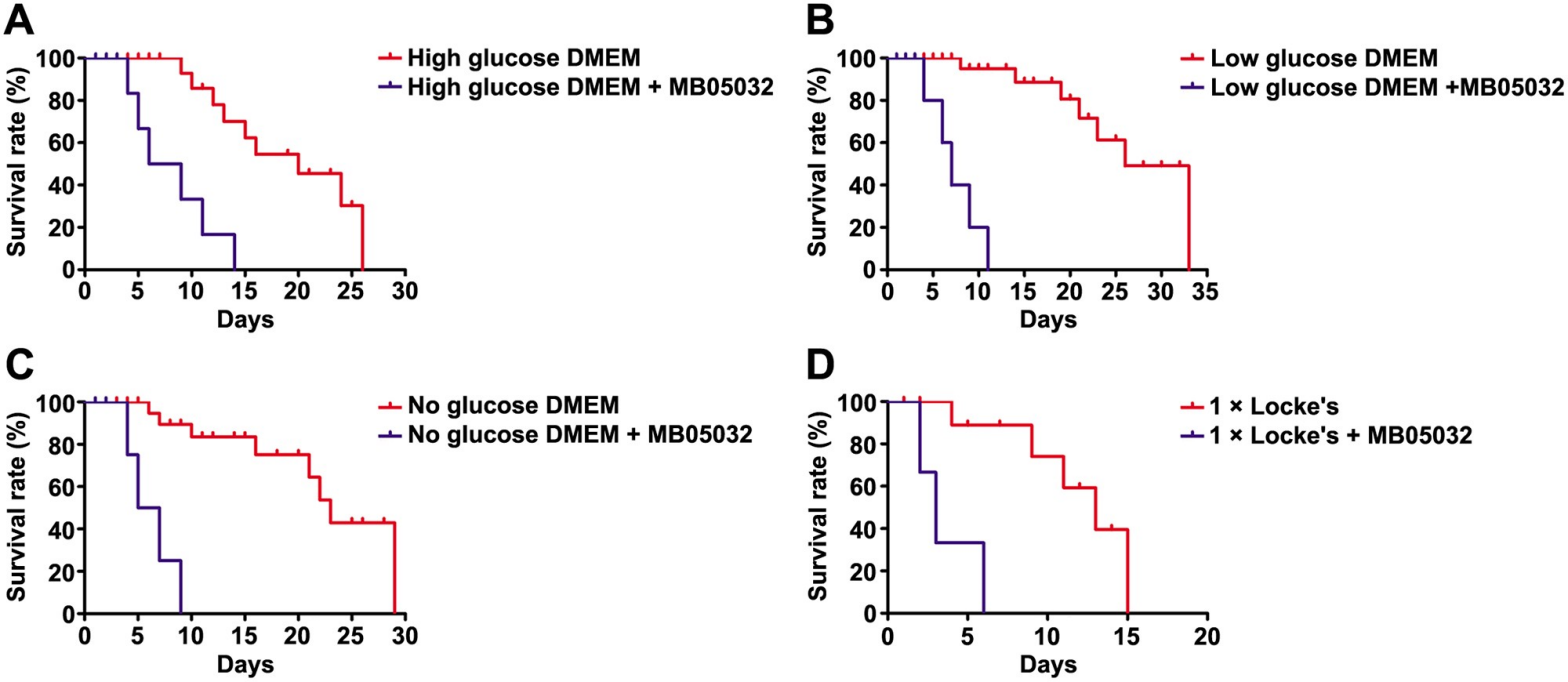
Survival curve of *C*. *sinensis* adults with or without MB05032 treatment. Adult flukes were respectively maintained on high, low, and no glucose DMEM (A, B, and C), as well as 1 × Locke’s (D) with or without the addition of MB05032. Survival curve of adult flukes was constructed using Kaplan-Meier analysis with the log-rank test.

MB05032 treatment sharply decreased the survival time of adult flukes in each culture solution (high glucose DMEM versus high glucose DMEM + MB05032, *P* < 0.0001; low glucose DMEM versus low glucose DMEM + MB05032, *P* < 0.0001; no glucose DMEM versus no glucose DMEM + MB05032, *P* < 0.0001; 1 × Locke’s versus 1 × Locke’s + MB05032, *P* < 0.01). Under high glucose DMEM condition, MB05032 treatment remarkably decreased median survival time from 20.0 ± 0.6 to 7.5 ± 0.2 d and maximum survival time from 26.2 ± 2.0 to 14.1 ± 1.7 d. Under low glucose DMEM condition, MB05032 treatment similarly decreased median survival time from 25.9 ± 1.0 to 7.0 ± 0.1 d and maximum survival time from 33.9 ± 3.0 to 12.0 ± 0.7 d. Under no glucose DMEM condition, MB05032 treatment also decreased median survival time from 23.0 ± 0.5 to 5.9 ± 0.1 d and maximum survival time from 29.3 ± 1.5 to 10.7 ± 0.3 d. Likewise, under 1 × Locke’s condition, remarkable decreases of median survival time from 13.0 ± 0.3 to 3.1 ± 0.1 d and maximum survival time from 17.5 ± 1.5 to 7.0 ± 0.1 d were observed with MB05032 treatment. Hence, in these solutions, MB05032 treatment showed a 62.5%-76.2% decrease in median survival time and a 46.2%-64.6% decrease in maximum survival time (Fig 9 and S4 Table).

## Discussion

Parasitic helminths have to adapt themselves to diverse environments in which oxygen, nutrients, temperature, and ion conditions vary considerably. Adequate energy sources are necessary for the parasite to grow, develop, reproduce, and long-term inhabit the host. Unlike adult schistosomes, who reside in a glucose-rich mesenteric vein [34], numerous *C*. *sinensis* adults are surrounded by a biliary environment, which seems unlikely to provide them with sufficient carbohydrates. The flukes may rely on other alternative sources in response to crowded dwelling and energy limitation. The present results showed that exogenous amino acids might be an essential energy source for *C*. *sinensis* adults to successfully survive in the host.

In the present study, the increase of total amino acids in *C*. *sinensis*-infected bile was in agreement with a previous study (Table 1) [35]. There might be two possible sources for these biliary amino acids. During *C*. *sinensis* infection, the moving and feeding flukes continuously rub the inner surface of bile ducts with their flexible body and puncture the small vessels with muscular suckers [36, 37]. Thus, many plasma amino acids, including Ser, Gly, Ala, His, Thr, and Lys, whose concentrations increased in *C*. *sinensis*-infected bile (Table 1), would be leaked from the injury of small vessels and blended into the bile [38]. The second possible source of these biliary amino acids might be the digestion of epithelial exfoliations and damaged tissues as well as plasma proteins from the host [35]. Emerging evidences have proven that several secreted proteases of trematodes, such as cysteine peptidases and aminopeptidases, can degrade host tissues, including hemoglobin, plasma, antibodies, and extracellular matrix, to small peptides [39–43]. The proteolytic cascade or network of these endopeptidases and exopeptidases, which constitute the major components of excretory-secretory products (ESPs), is presumed to hydrolyze host tissues into free amino acids [44–47]. The constant production of extracorporeal amino acids might be of considerable significance reflecting the nutrients of *C*. *sinensis* adults, supported by the increase of urea concentration in *C*. *sinensis*-infected bile (Table 1). Urea nitrogen from the catabolism of amino acids serves as a reliable indicator of dietary protein intake [48]. It has been documented that the excretion of urea is a basic metabolic pathway of *Fasciola hepatica* and *Fasciola gigantica* for removal of highly toxic ammonium ions from the catabolism of amino acids [49, 50]. Therefore, urea dramatically increased in *C*. *sinensis*-infected bile was most likely excreted from *C*. *sinensis* adults in the same way, implying that the uptake and catabolism of amino acids might occur in their bodies. In *C*. *sinensis*-infected bile, since 6 of the 9 enriched amino acids are glycogenic amino acids (Table 1), they might not only provide nitrogenous demand for adult flukes to maintain body structure, but also serve as a fuel for their growth and development. The concentrations of Lys and Thr, which are respectively ketogenic as well as glycogenic and ketogenic amino acids, also increased in *C*. *sinensis*-infected bile (Table 1). This result suggested that ketone bodies might be another energy source when the flukes undergo insufficiency of carbohydrate or longstanding starvation. Further studies will be necessary to confirm the contribution of ketone bodies to energy metabolism of *C*. *sinensis* adults. It is unclear why carnosine was presented in *C*. *sinensis*-infected bile (Table 1). Carnosine, which is an endogenous dipeptide widely distributed in the muscle and nervous tissues of vertebrates, exerts many biological functions, including antioxidant activity, antitoxic activity, antiinflammatory activity, and neuroprotective activity [51]. Thus, carnosine is predicted to be released from host cells in response to chronic stimulation from *C*. *sinensis* ESPs associated with free radical generation [52]. Carnosine possibly functions as an antioxidant and scavenger of free radicals.

Gly and Ala are two of the most abundant amino acids in plasma. Since both of them were increased in *C*. *sinensis-*infected bile [38] (Table 1), we speculated that they could be utilized by adult flukes to produce energy. As expected, Gly + Ala and glycerol feeding remarkably prolonged the survival time of adult flukes (Fig 1A and S2 Table), suggesting that these nutrients are indispensable for ATP production during the absence of glucose. In humans, the conversion of Gly and Ala to glucose via gluconeogenesis is controlled by the catalysis of G-6-Pase, FBPase, PC, and PEPCK, while the conversion of glycerol to glucose via gluconeogenesis is controlled by the catalysis of G-6-Pase and FBPase [16]. The results that Gly + Ala feeding upregulated the expression levels of *Cs*G-6-Pase, *Cs*FBPase, *Cs*PEPCK, and *Cs*PC, but glycerol feeding did not upregulate the expression levels of *Cs*PEPCK and *Cs*PC (Fig 2A–2D), indicated similar control steps in the gluconeogenesis pathway of *C*. *sinensis* adults. This kind of evolution might be beneficial for energy metabolism of adult flukes. Under Gly + Ala condition, the upregulation of *Cs*G-6-Pase, *Cs*FBPase, *Cs*PEPCK, and *Cs*PC expression at 3, 5, and 7 d reflected a strong gluconeogenesis rate, with continuous conversion of exogenous Gly and Ala to glucose (Fig 2A–2D). Consistent with this finding, the four rate-limiting enzymes were expressed increasingly over time (Fig 2E–2H). As to glycerol condition, the upregulation of *Cs*G-6-Pase and *Cs*FBPase expression only at 3 d reflected a transient conversion of glycerol into glucose inside the body of adult flukes (Fig 2A and 2B). These results suggest that exogenous Gly and Ala appeared to be more important for *C*. *sinensis* adults than exogenous glycerol in the absence of glucose.

Trematodes have the ability to import glucose and amino acids directly across their tegument facilitated by glucose and amino acid transporters [34, 53–55]. Thus, exogenous glucose and amino acids in DMEM could be absorbed and catabolized by *C*. *sinensis* adults, which is coupled to ATP production for extending the survival time of adult flukes [12, 18] (Fig 1B and S3 Table). However, neither high nor low glucose DMEM could provide more advantageous conditions to extend the survival time of adult flukes than no glucose DMEM (Fig 1B and S3 Table), implying that exogenous amino acids alone would meet energy requirements for the survival of adult flukes. Because of the lack of nutrients in 1 × Locke’s, gluconeogenesis might take place inside the body of adult flukes for rapid replenishment of glucose [56]. Thus, it seems reasonable that the highest expression levels of *Cs*G-6-Pase, *Cs*FBPase, *Cs*PEPCK, and *C*sPC were in 1 × Locke’s but not in no glucose DMEM at 3 d (Fig 3A–3D). Thereafter, the highest expression levels of *Cs*G-6-Pase, *Cs*FBPase, *Cs*PEPCK, and *C*sPC were in no glucose DMEM (Fig 3A–3D). In no glucose DMEM, the progressively enhanced expression levels of the four rate-limiting enzymes indicated a gradually enhanced gluconeogenesis rate (Fig 3E–3H), suggesting that glucose could be constantly replenished through the transformation of exogenous amino acids in the absence of glucose. In addition, although adult flukes failed to express higher levels of the four rate-limiting enzymes in high and low glucose DMEM than those expressed in 1 × Locke’s (Fig 3A–3D), it is unreasonable to deny the consumption of exogenous amino acids in these DMEM. Hence, we next detected the L-amino acid level in cultured supernatants. Intriguingly, L-amino acid levels gradually reduced in high, low, and no glucose DMEM, suggesting the ingestion of exogenous amino acids by adult flukes regardless of the absence or presence of glucose (Fig 4). It was reported that exogenous amino acids could be utilized by *C*. *sinensis* adults not only for synthesis of the protein, but also for energy production. By comparing with the catabolism of glucose, the researchers proposed that respiratory CO_2_ from ^14^C-glycine was greater than that from ^14^C-glucose in unit wet weight of the worms [57]. This finding hinted at the possibility that adult flukes could obtain a large amount of energy from amino acids. Similar to other trematodes, *C*. *sinensis* adults can acquire nutrients, such as amino acids and glucose, by feeding on host tissue and blood [58, 59]. Since the normal blood glucose level of humans changes with the diet and is no more than 10 mM, adult flukes seem unlikely to acquire more glucose from the bile ducts of their host [60]. Therefore, our finding that adult flukes survived for less time in high glucose DMEM (25 mM glucose) than those in low glucose DMEM survived (5.6 mM glucose) might be attributed to their adaptation to the limited-glucose bile ducts (Fig 1B and S3 Table). These results seemed to partially reflect the metabolic characteristics of *C*. *sinensis* adults, showing that adult flukes possibly not only rely on glucose from the host, but also require exogenous amino acids for energy production.

In mammals, gluconeogenesis plays a crucial role in maintaining glucose homeostasis [61]. After a few hours of fasting, glycogen stores become exhausted and then the supply of blood glucose relies entirely on its biosynthesis from non-carbohydrate stores through gluconeogenesis [16]. Since FBPase regulates the control step in gluconeogenesis, suppression of FBPase activity will theoretically block glucose synthesis. Unlike the natural inhibitor AMP, MB05032 exhibits high specificity and potency for human FBPase. It has an efficacious effect on the reduction of glucose synthesis from some common glycogenic precursors in rat and human hepatocytes [28]. Considering that *Cs*FBPase shares 62% identity with human FBPase and possesses a similar FBPase motif by BLASTx analysis [62], the activities of r*Cs*FBPase and native *Cs*FBPase could also be effectively inhibited by MB05032 (S2 Fig). Furthermore, accompanying with the inhibition of native *Cs*FBPase activity (Fig 5), the glucose level inside the body of adult flukes was remarkably decreased (Fig 6). This result suggested that MB05032 could inhibit the production of glucose through gluconeogenesis by targeting *Cs*FBPase. Almost all living helminths in anaerobic and semi-aerobic habitats require carbohydrates to produce energy and metabolites [12]. Once in the absence of glucose, *schistosomes* rapidly degrade glycogen stores for survival [63]. Based on the distribution of glycogen in *Fasciola hepatica* [64], glycogen stores of *C*. *sinensis* adults are supposed to lie within the yolk cell, peripheral muscle, parenchyma layer, and fine tonofibril of tegument. When gluconeogenesis was inhibited, glycogen stores might be gradually degraded by *C*. *sinensis* adults for maintenance of carbohydrate supply [65], which resulted in the obvious decrease of glycogen levels after MB05032 treatment (Fig 7). Accordingly, the decreased activity and viability of adult flukes might be related to the exhaustion of glucose and glycogen by the inhibition of gluconeogenesis (Figs 8 and 9, S1–S8 Videos and S4 Table). This dramatic effect indicates that gluconeogenesis may play a vital role in maintaining glucose homeostasis for the survival of adult flukes under an adverse condition. These observations support above results, revealing that exogenous amino acids might be essential for adult flukes regardless of the absence or presence of exogenous glucose. Accordingly, it is possible that *C*. *sinensis* adults absorb exogenous amino acids for energy production even if they can obtain glucose from the host. In addition to their role in energy metabolism, amino acids are possibly involved in maintenance of body structure, formation of new protoplasm, and regulation of intracellular osmotic pressure during the host-helminth coexisting period [66]. Further studies are needed to investigate whether exogenous amino acids participate in other physiological processes of *C*. *sinensis* adults.

In summary, our current results suggest that exogenous amino acids probably serve as an important energy source that benefits the continued survival of *C*. *sinensis* adults, thus providing a valuable strategy for design of new anthelmintics against the gluconeogenesis pathway of *C*. *sinensis*. Our foundational study opens a new avenue for illuminating energy metabolism and parasitic mechanism of *C*. *sinensis*.

## Supporting information

S1 Fig Detection of free amino acids in 20 μl of the bile sample. (A) A *C*. *sinensis*-infected bile sample. (B) An uninfected bile sample. (C) The standard sample. Concentration of free amino acids in bile samples was calculated by using the standard curves generated by the standard sample.
(PDF)

S2 Fig Specific inhibition of r*Cs*FBPase and native *Cs*FBPase by MB05032. (A) Percent inhibition of r*Cs*FBPase by 0–50 mM AMP or 0–500 μM MB05032. (B) Percent inhibition of native *Cs*FBPase in soluble protein of adult flukes by 0–50 mM AMP or 0–500 μM MB05032. (C) Inhibition of r*Cs*FBPase and r*Cs*FbA-2 by 10 μM MB05032. The 50% inhibiting concentration (IC50) of AMP (positive control) and MB05032 against r*Cs*FBPase or native *Cs*FBPase were calculated using four-parameter logistics nonlinear regression, respectively. The activities of r*Cs*FBPase or r*Cs*FbA-2 with or without the addition of MB05032 were compared by student’s *t* test. **P < 0.01 was represented as statistical significance; n.s., not significant.
(PDF)

S1 Video Morphological and moving changes of *C*. *sinensis* adults maintained on high glucose DMEM. (AVI)

S2 Video Morphological and moving changes of *C*. *sinensis* adults maintained on high glucose DMEM with MB05032 treatment. (AVI)

S3 Video Morphological and moving changes of *C*. *sinensis* adults maintained on low glucose DMEM. (AVI)

S4 Video Morphological and moving changes of *C*. *sinensis* adults maintained on low glucose DMEM with MB05032 treatment. (AVI)

S5 Video Morphological and moving changes of *C*. *sinensis* adults maintained on no glucose DMEM. (AVI)

S6 Video Morphological and moving changes of *C*. *sinensis* adults maintained on no glucose DMEM with MB05032 treatment. (AVI)

S7 Video Morphological and moving changes of *C*. *sinensis* adults maintained on 1 × Locke’s. (AVI)

S8 Video Morphological and moving changes of *C*. *sinensis* adults maintained on 1 × Locke’s with MB05032 treatment. (AVI)

S1 Table Primers used in qRT-PCR. (PDF)

S2 Table Survival time of *C*. *sinensis* adults maintained on 1 × Locke’s with or without the addition of different nutrients. Survival time was evaluated by the log-rank test. *P* < 0.05 and *P* < 0.01 were represented as statistical significance.
(PDF)

S3 Table Survival time of *C*. *sinensis* adults maintained on 1 × Locke’s and different DMEM. Survival time was evaluated by the log-rank test. *P* < 0.05 and *P* < 0.01 were represented as statistical significance.
(PDF)

S4 Table Survival time of *C*. *sinensis* adults with or without MB05032 treatment. Survival time was evaluated by the log-rank test. *P* < 0.01 and *P* < 0.0001 were represented as statistical significance.
(PDF)

## References

[1] Lun ZR, Gasser RB, Lai DH, Li AX, Zhu XQ, Yu XB, et al Clonorchiasis: a key foodborne zoonosis in China. Lancet Infect Dis. 2005; 5(1): 31–41. 10.1016/S1473-3099(04)01252-6 15620559

[2] Keiser J, Utzinger J. Food-borne trematodiases. Clin Microbiol Rev. 2009; 22(3): 466–483. 10.1128/CMR.00012-09 19597009 PMC2708390

[3] Lim JH. Liver flukes: the malady neglected. Korean J Radiol. 2011; 12(3): 269–279. 10.3348/kjr.2011.12.3.269 21603286 PMC3088844

[4] Shin HR, Oh JK, Masuyer E, Curado MP, Bouvard V, Fang YY, et al Epidemiology of cholangiocarcinoma: an update focusing on risk factors. Cancer Sci. 2010; 101(3): 579–585. 10.1111/j.1349-7006.2009.01458.x 20085587 PMC11158235

[5] Bouvard V, Baan R, Straif K, Grosse Y, Secretan B, El Ghissassi F, et al A review of human carcinogens—Part B: biological agents. Lancet Oncol. 2009; 10(4): 321–322. 10.1016/s1470-2045(09)70096-8 19350698

[6] de Martel C, Ferlay J, Franceschi S, Vignat J, Bray F, Forman D, et al Global burden of cancers attributable to infections in 2008: a review and synthetic analysis. Lancet Oncol. 2012; 13(6): 607–615. 10.1016/S1470-2045(12)70137-7 22575588

[7] Correia da Costa JM, Vale N, Gouveia MJ, Botelho MC, Sripa B, Santos LL, et al Schistosome and liver fluke derived catechol-estrogens and helminth associated cancers. Front Genet. 2014; 5: 444 10.3389/fgene.2014.00444 25566326 PMC4274992

[8] Lee JM, Lim HS, Hong ST. Hypersensitive reaction to praziquantel in a clonorchiasis patient. Korean J Parasitol. 2011; 49(3): 273–275. 10.3347/kjp.2011.49.3.273 22072827 PMC3210844

[9] Kyung SY, Cho YK, Kim YJ, Park JW, Jeong SH, Lee JI, et al A paragonimiasis patient with allergic reaction to praziquantel and resistance to triclabendazole: successful treatment after desensitization to praziquantel. Korean J Parasitol. 2011; 49(1): 73–77. 10.3347/kjp.2011.49.1.73 21461273 PMC3063930

[10] Tinga N, De N, Vien HV, Chau L, Toan ND, Kager PA, et al Little effect of praziquantel or artemisinin on clonorchiasis in Northern Vietnam. A pilot study. Trop Med Int Health. 1999; 4(12): 814–818. 10.1046/j.1365-3156.1999.00499x 10632989

[11] Xu LL, Jiang B, Duan JH, Zhuang SF, Liu YC, Zhu SQ, et al Efficacy and Safety of Praziquantel, Tribendimidine and Mebendazole in Patients with Co-infection of Clonorchis sinensis and Other Helminths. PLoS Negl Trop Dis. 2014; 8(8): e3046 10.1371/journal.pntd.0003046 25122121 PMC4133228

[12] Kang IK, Lee SH, Seo BS. Study on the (14)C-glucose metabolism by Clonorchis sinensis: Paper Chromatographic Analyses in Combination with Autoradiography. Kisaengchunghak Chapchi. 1969; 7(3): 143–152. 10.3347/kjp.1967.7.3.143 12913527

[13] van Vugt F, van der Meer P, van den Bergh SG. The formation of propionate and acetate as terminal processes in the energy metabolism of the adult liver fluke Fasciola hepatica. Int J Biochem. 1979; 10(1): 11–18. 10.1016/0020-711x(79)90133-2 421954

[14] Huang Y, Chen W, Wang X, Liu H, Chen Y, Guo L, et al The carcinogenic liver fluke, Clonorchis sinensis: new assembly, reannotation and analysis of the genome and characterization of tissue transcriptomes. PLoS One. 2013; 8(1): e54732 10.1371/journal.pone.0054732 23382950 PMC3559784

[15] Snell K. Muscle alanine synthesis and hepatic gluconeogenesis. Biochem Soc Trans. 1980; 8(2): 205–213. 10.1042/bst0080205 6989679

[16] Hers HG, Hue L. Gluconeogenesis and related aspects of glycolysis. Annu Rev Biochem. 1983; 52: 617–653. 10.1146/annurev.bi.52.070183.003153 6311081

[17] Wang X, Chen W, Huang Y, Sun J, Men J, Liu H, et al The draft genome of the carcinogenic human liver fluke Clonorchis sinensis. Genome Biol. 2011; 12(10): R107 10.1186/gb-2011-12-10-r107 22023798 PMC3333777

[18] Seong SH, Seo BS. Metabolism of C(14)-glycine by Clonorchis sinensis. Kisaengchunghak Chapchi. 1966; 4(2): 14–22. 10.3347/kjp.1966.4.2.14 12913568

[19] Yoon JS. Studies On The Metabolism Of C(14)-Proline In Some Parasitic Helminths. Kisaengchunghak Chapchi. 1964; 2(3): 159–164. 10.3347/kjp.1964.2.3.159 12913596

[20] Han SS. Autoradiographic Studies On The Uptake Of (14)C-Succinic Acid By Clonorchis Sinensis. Kisaengchunghak Chapchi. 1971; 9(1): 17–24. 10.3347/kjp.1971.9.1.17 12913621

[21] Hahn HJ. Utilization of C14 Labeled Acetate into Respiratory Carbon Dioxide, Glycogen, Lipids and Protein Fractions by Clonorchis Sinensis in Vitro. Taehan Naekwa Hakhoe Chapchi. 1963; 143: 377–382. 14083288

[22] Fang-Qi O, Zi-Yue W, Hai-Yan W, Yao-Bao W, Yi-Chao Y, Yun-Liang S. Investigation on Clonorchis sinensis infections in marketed cats in Nanning City. Zhongguo Xue Xi Chong Bing Fang Zhi Za Zhi. 2019; 31(3): 299–300. 10.16250/j.32.1374.2018281 31544411

[23] Liang C, Hu XC, Lv ZY, Wu ZD, Yu XB, Xu J, et al Experimental establishment of life cycle of Clonorchis sinensis. Zhongguo Ji Sheng Chong Xue Yu Ji Sheng Chong Bing Za Zhi. 2009; 27(2): 148–150. 19856506

[24] Uddin MH, Bae YM, Choi MH, Hong ST. Production and deformation of Clonorchis sinensis eggs during in vitro maintenance. PLoS One. 2012; 7(12): e52676 10.1371/journal.pone.0052676 23285144 PMC3527588

[25] Uddin MH, Li S, Bae YM, Choi MH, Hong ST. In vitro maintenance of clonorchis sinensis adult worms. Korean J Parasitol. 2012; 50(4): 309–315. 10.3347/kjp.2012.50.4.309 23230328 PMC3514422

[26] Yoo WG, Kim TI, Li S, Kwon OS, Cho PY, Kim TS, et al Reference genes for quantitative analysis on Clonorchis sinensis gene expression by real-time PCR. Parasitol Res. 2009; 104(2): 321–328. 10.1007/s00436-008-1195-x 18815810

[27] Livak KJ, Schmittgen TD. Analysis of relative gene expression data using real-time quantitative PCR and the 2(-Delta Delta C(T)) Method. Methods. 2001; 25(4): 402–408. 10.1006/meth.2001.1262 11846609

[28] Erion MD, van Poelje PD, Dang Q, Kasibhatla SR, Potter SC, Reddy MR, et al MB06322 (CS-917): A potent and selective inhibitor of fructose 1,6-bisphosphatase for controlling gluconeogenesis in type 2 diabetes. Proc Natl Acad Sci U S A. 2005; 102(22): 7970–7975. 10.1073/pnas.0502983102 15911772 PMC1138262

[29] Liang P, Sun J, Huang Y, Zhang F, Zhou J, Hu Y, et al Biochemical characterization and functional analysis of fructose-1,6-bisphosphatase from Clonorchis sinensis. Mol Biol Rep. 2013; 40(7): 4371–4382. 10.1007/s11033-013-2508-4 23652997

[30] Inoue T, Yatsuki H, Kusakabe T, Joh K, Takasaki Y, Nikoh N, et al Caenorhabditis elegans has two isozymic forms, CE-1 and CE-2, of fructose-1,6-bisphosphate aldolase which are encoded by different genes. Arch Biochem Biophys. 1997; 339(1): 226–234. 10.1006/abbi.1996.9813 9056253

[31] Gidh-Jain M, Zhang Y, van Poelje PD, Liang JY, Huang S, Kim J, et al The allosteric site of human liver fructose-1,6-bisphosphatase. Analysis of six AMP site mutants based on the crystal structure. J Biol Chem. 1994; 269(44): 27732–27738. 7961695

[32] Li S, Bian M, Wang X, Chen X, Xie Z, Sun H, et al Molecular and biochemical characterizations of three fructose-1,6-bisphosphate aldolases from Clonorchis sinensis. Mol Biochem Parasitol. 2014; 194(1–2): 36–43. 10.1016/j.molbiopara.2014.04.005 24768689

[33] Lee SJ, Murphy CT, Kenyon C. Glucose shortens the life span of C. elegans by downregulating DAF-16/FOXO activity and aquaporin gene expression. Cell Metab. 2009; 10(5): 379–391. 10.1016/j.cmet.2009.10.003 19883616 PMC2887095

[34] Krautz-Peterson G, Simoes M, Faghiri Z, Ndegwa D, Oliveira G, Shoemaker CB, et al Suppressing glucose transporter gene expression in schistosomes impairs parasite feeding and decreases survival in the mammalian host. PLoS Pathog. 2010; 6(6): e1000932 10.1371/journal.ppat.1000932 20532163 PMC2880588

[35] Isseroff H, Tunis M, Read CP. Changes in amino acids of bile in Fasciola hepatica infections. Comparative Biochemistry and Physiology. B: Comparative Biochemistry. 1972; 41(1): 157–163. 10.1016/0305-0491(72)90018-1 5075380

[36] Rim HJ. The current pathobiology and chemotherapy of clonorchiasis. Kisaengchunghak Chapchi. 1986; 24 Suppl: 1–141. 10.33447/kjp.1986.24.suppl.1 12902642

[37] Hou PC. The pathology of Clonorchis sinensis infestation of the liver. J Pathol Bacteriol. 1955; 70(1): 53–64. 10.1002/path.1700700106 13272122

[38] Felig P, Marliss E, Pozefsky T, Cahill GF Jr. Amino acid metabolism in the regulation of gluconeogenesis in man. Am J Clin Nutr. 1970; 23(7): 986–992. 10.1093/ajcn/23.7.986 5455559

[39] Cortes A, Mikes L, Munoz-Antoli C, Alvarez-Izquierdo M, Esteban JG, Horak P, et al Secreted cathepsin L-like peptidases are involved in the degradation of trapped antibodies on the surface of Echinostoma caproni. Parasitology Research. 2019; 118(12): 3377–3386. 10.1007/s00436-019-06487-4 31720841

[40] Berasain P, Carmona C, Frangione B, Dalton JP, Goni F. Fasciola hepatica: parasite-secreted proteinases degrade all human IgG subclasses: determination of the specific cleavage sites and identification of the immunoglobulin fragments produced. Experimental Parasitology. 2000; 94(2): 99–110. 10.1006/expr.1999.4479 10673346

[41] Berasain P, Goni F, McGonigle S, Dowd A, Dalton JP, Frangione B, et al Proteinases secreted by Fasciola hepatica degrade extracellular matrix and basement membrane components. Journal of Parasitology. 1997; 83(1): 1–5. 9057688

[42] Sripa J, Laha T, To J, Brindley PJ, Sripa B, Kaewkes S, et al Secreted cysteine proteases of the carcinogenic liver fluke, Opisthorchis viverrini: regulation of cathepsin F activation by autocatalysis and trans-processing by cathepsin B. Cell Microbiol. 2010; 12(6): 781–795. 10.1111/j.1462-5822.2010.01433.x 20070308 PMC2888773

[43] Deng C, Sun J, Li X, Wang L, Hu X, Wang X, et al Molecular identification and characterization of leucine aminopeptidase 2, an excretory-secretory product of Clonorchis sinensis. Molecular Biology Reports. 2012; 39(10): 9817–9826. 10.1007/s11033-012-1848-9 22729885

[44] Na BK, Kang JM, Sohn WM. CsCF-6, a novel cathepsin F-like cysteine protease for nutrient uptake of Clonorchis sinensis. International Journal for Parasitology. 2008; 38(5): 493–502. 10.1016/j.ijpara.2007.09.001 17945236

[45] Siricoon S, Vichasri Grams S, Lertwongvisarn K, Abdullohfakeeyah M, Smooker PM, Grams R. Fasciola gigantica cathepsin B5 is an acidic endo- and exopeptidase of the immature and mature parasite. Biochimie. 2015; 119: 6–15. 10.1016/j.biochi.2015.10.005 26453811

[46] Kang JM, Lee J, Ju HL, Ju JW, Kim JH, Pak JH, et al Characterization of a gut-associated asparaginyl endopeptidase of Clonorchis sinensis. Experimental Parasitology. 2015; 153: 81–90. 10.1016/j.exppara.2015.03.015 25819296

[47] Delcroix M, Sajid M, Caffrey CR, Lim KC, Dvorak J, Hsieh I, et al A multienzyme network functions in intestinal protein digestion by a platyhelminth parasite. Journal of Biological Chemistry. 2006; 281(51): 39316–39329. 10.1074/jbc.M607128200 17028179

[48] Simmons WK. Urinary urea nitrogen-creatinine ratio as indicator of recent protein intake in field studies. Am J Clin Nutr. 1972; 25(5): 539–542. 10.1093/ajcn/25.5.539 4553789

[49] Kurelec B. Catabolic path of arginine and NAD regeneration in the parasite Fasciola hepatica. Comp Biochem Physiol B. 1975; 51(2): 151–156. 10.1016/0305-0491(75)90199-6 166790

[50] Mohamed SA, Fahmy AS, Mohamed TM, Hamdy SM. Urea cycle of Fasciola gigantica: purification and characterization of arginase. Comp Biochem Physiol B Biochem Mol Biol. 2005; 142(3): 308–316. 10.1016/j.cbpb.2005.08.002 16125991

[51] Budzen S, Rymaszewska J. The biological role of carnosine and its possible applications in medicine. Adv Clin Exp Med. 2013; 22(5): 739–744. 24285460

[52] Pak JH, Moon JH, Hwang SJ, Cho SH, Seo SB, Kim TS. Proteomic analysis of differentially expressed proteins in human cholangiocarcinoma cells treated with Clonorchis sinensis excretory-secretory products. J Cell Biochem. 2009; 108(6): 1376–1388. 10.1002/jcb.22368 19798681

[53] Ahn SK, Cho PY, Na BK, Hong SJ, Nam HW, Sohn WM, et al Molecular cloning and functional characterization of a glucose transporter (CsGLUT) in Clonorchis sinensis. Parasitology Research. 2016; 115(1): 347–354. 10.1007/s00436-015-4754-y 26450594

[54] McKenzie M, Kirk RS, Walker AJ. Glucose Uptake in the Human Pathogen Schistosoma mansoni Is Regulated Through Akt/Protein Kinase B Signaling. Journal of Infectious Diseases. 2018; 218(1): 152–164. 10.1093/infdis/jix654 29309602 PMC5989616

[55] Krautz-Peterson G, Camargo S, Huggel K, Verrey F, Shoemaker CB, Skelly PJ. Amino acid transport in schistosomes: Characterization of the permeaseheavy chain SPRM1hc. Journal of Biological Chemistry. 2007; 282(30): 21767–21775. 10.1074/jbc.M703512200 17545149

[56] Coles GC. Recent advances in schistosome biochemistry. Parasitology. 1984; 89 (Pt 3): 603–637. 10.1017/s0031182000056808 6393006

[57] Seong SH, Seo BS. Metabolism of C(14)-glycine by Clonorchis sinensis. Kisaengchunghak Chapchi. Korean Journal of Parasitology. 1966; 4(2): 14–22. 10.3347/kjp.1966.4.2.14 12913568

[58] Isseroff H, Read CP. Studies on membrane transport. VI. Absorption of amino acids by fascioliid trematodes. Comparative Biochemistry and Physiology. 1969; 30(6): 1153–1159. 10.1016/0010-406x(69)91050-0 5349637

[59] Todd JR, Ross JG. Observations on the use of radioactive 51Cr-labelled red cells to study anaemia in Fasciola hepatica infections in cattle. Clinica Chimica Acta. 1966; 14(1): 27–30. 10.1016/0009-8981(66)90060-x 6006200

[60] Alberti KG, Zimmet PZ. Definition, diagnosis and classification of diabetes mellitus and its complications. Part 1: diagnosis and classification of diabetes mellitus provisional report of a WHO consultation. Diabetic Medicine. 1998; 15(7): 539–553. 10.1002/(SICI)1096-9136(199807)15:7<539::AID-DIA668>3.0.CO;2-S 9686693

[61] Groen AK, Vervoorn RC, Van der Meer R, Tager JM. Control of gluconeogenesis in rat liver cells. I. Kinetics of the individual enzymes and the effect of glucagon. J Biol Chem. 1983; 258(23): 14346–14353. 6643485

[62] Zheng M, Hu K, Liu W, Hu X, Hu F, Huang L, et al Proteomic analysis of excretory secretory products from Clonorchis sinensis adult worms: molecular characterization and serological reactivity of a excretory-secretory antigen-fructose-1,6-bisphosphatase. Parasitology Research. 2011; 109(3): 737–744. 10.1007/s00436-011-2316-5 21424807

[63] Bueding E, Koletsky S. Content and distribution of glycogen in Schistosoma mansoni. Proceedings of the Society for Experimental Biology and Medicine. 1950; 73(4): 594–596. 10.3181/00379727-73-17755 15417608

[64] Pantelouris EM. Localization of Glycogen in Fasciola Hepatica L. And an Effect of Insulin. Journal of Helminthology. 1964; 38: 283–286. 10.1017/s0022149x00033848 14250814

[65] Halton DW. Nutritional adaptations to parasitism within the platyhelminthes. Int J Parasitol. 1997; 27(6): 693–704. 10.1016/s0020-7519(97)00011-8 9229252

[66] Barrett J. Amino acid metabolism in helminths. Adv Parasitol. 1991; 30: 39–105. 10.1016/s0065-308x(08)60306-1 2069074

