# Supplementary figures and images for: Amino acids serve as an important energy source for adult flukes of Clonorchis sinensis
Source: PLoS Negl Trop Dis. 2020 Apr 30;14(4):e0008287. doi: 10.1371/journal.pntd.0008287 (PMC7217481; doi:10.1371/journal.pntd.0008287)

**A**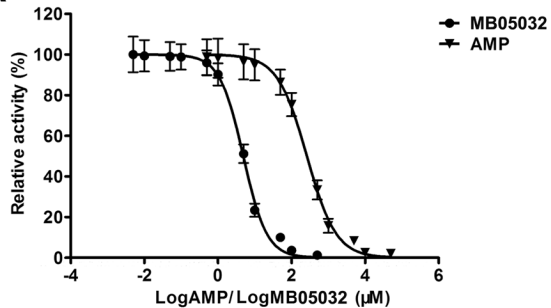**B**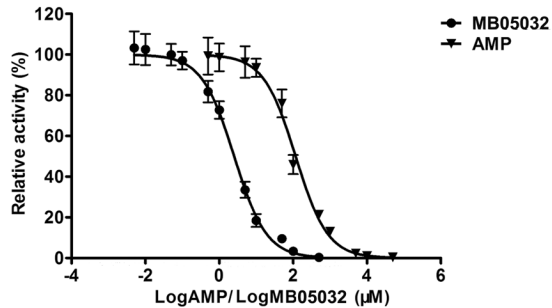**C**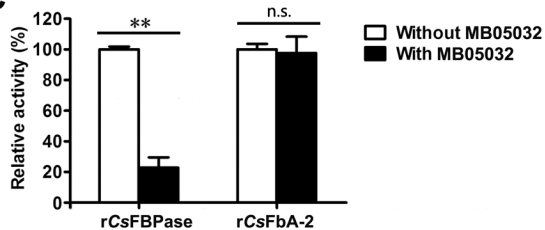

Supplement: S2 Fig — (A) Percent inhibition of rCsFBPase by 0–50 mM AMP or 0–500 μM MB05032. (B) Percent inhibition of native CsFBPase in soluble protein of adult flukes by 0–50 mM AMP or 0–500 μM MB05032. (C) Inhibition of rCsFBPase and rCsFbA-2 by 10 μM MB05032. The 50% inhibiting concentration (IC50) of AMP (positive control) and MB05032 against rCsFBPase or native CsFBPase were calculated using four-parameter logistics nonlinear regression, respectively. The activities of rCsFBPase or rCsFbA-2 with or without the addition of MB05032 were compared by student’s t test. **P < 0.01 was represented as statistical significance; n.s., not significant. (PDF) [file pntd.0008287.s002.pdf]
