# Supplementary material for: Amino acids serve as an important energy source for adult flukes of Clonorchis sinensis
Source: PLoS Negl Trop Dis. 2020 Apr 30;14(4):e0008287. doi: 10.1371/journal.pntd.0008287 (PMC7217481; doi:10.1371/journal.pntd.0008287)
Supplement: S1 Table — (PDF) [file pntd.0008287.s011.pdf]

S1 Table. Primers used in qRT-PCR.

	**Forward primer**	**Reverse primer**
***Cs*G-6-Pase (DF144183.1)**	5'-CTTGCCGGAGTACTTTCAGGCC-3'	5'-AAGCCGATCAACAGACCAGGCG-3'
***Cs*FBPase (DF144433.1)**	5'-TCTATGGTAGTGCGACAGTGGTGG-3'	5'-CCTGATCCCAGAGTGATGCGTAG-3'
***Cs*PEPCK (DF142981.1)**	5'-TTTCATCAAAGCCCTCCATTC-3'	5'-ATGTGCGAGATCAAGGTCAGC-3'
***Cs*PC (DF144082.1)**	5'-CTCAGCGAGAGGTCAGACTTTGC-3'	5'-TTCCACGGTAGTAACTGGTCCGG-3'
***C. sinensis* β-actin (EU109284.1)**	5'-ACCGTGAGAAGATGACGCAGA-3'	5'-GCCAAGTCCAAACGAAGAATT-3'
