# Supplementary material for: Amino acids serve as an important energy source for adult flukes of Clonorchis sinensis
Source: PLoS Negl Trop Dis. 2020 Apr 30;14(4):e0008287. doi: 10.1371/journal.pntd.0008287 (PMC7217481; doi:10.1371/journal.pntd.0008287)
Supplement: S2 Table — Survival time was evaluated by the log-rank test. P < 0.05 and P < 0.01 were represented as statistical significance. (PDF) [file pntd.0008287.s012.pdf]

S2 Table. Survival time of *C. sinensis* adults maintained on 1 × Locke's with or without the addition of different nutrients.

	Median survival time (days ± SD)	Max survival time (days ± SD)	*P* value		
			**Gly + Ala[a]**	**Glycerol[b]**	**Control[c]**
**Gly + Ala[a]**	19.1 ± 1.7	25.3 ± 1.5	-	n.s. (0.4161)[d]	< 0.01 (0.0016)[e]
**Glycerol[b]**	17.0 ± 1.5	24.2 ± 1.0	n.s. (0.4161)[d]	-	< 0.05 (0.0145)[f]
**Control[c]**	10.5 ± 0.5	17.8 ± 0.8	< 0.01 (0.0016)[e]	< 0.05 (0.0145)[f]	-

[a]1 × Locke's containing 0.25% Gly and 0.25% Ala.

[b]1 × Locke's containing 0.5% glycerol.

[c]1 × Locke's.

[d]Gly + Ala versus glycerol.

[e]Gly + Ala versus control.

[f]Glycerol versus control.
