# Supplementary material for: Amino acids serve as an important energy source for adult flukes of Clonorchis sinensis
Source: PLoS Negl Trop Dis. 2020 Apr 30;14(4):e0008287. doi: 10.1371/journal.pntd.0008287 (PMC7217481; doi:10.1371/journal.pntd.0008287)
Supplement: S3 Table — Survival time was evaluated by the log-rank test. P < 0.05 and P < 0.01 were represented as statistical significance. (PDF) [file pntd.0008287.s013.pdf]

S3 Table. Survival time of *C. sinensis* adults maintained on 1 × Locke's and different DMEM.

	Median survival time (days ± SD)	Max survival time (days ± SD)	***P* value**			
			**High glucose DMEM**	**Low glucose DMEM**	**No glucose DMEM**	**1 × Locke's**
**High glucose DMEM**	20.0 ± 1.0	25.8 ± 1.5	-	< 0.05 (0.0361)[a]	n.s. (0.0974)[b]	< 0.05 (0.0487)[c]
**Low glucose DMEM**	27.1 ± 1.5	32.2 ± 2.5	< 0.05 (0.0361) [a]	-	n.s. (0.6954)[d]	< 0.01 (0.0003)[e]
**No glucose DMEM**	24.9 ± 1.1	30.0 ± 2.0	n.s. (0.0974)[b]	n.s. (0.6954)[d]	-	< 0.01 (0.0028)[f]
**1 × Locke's**	12.0 ± 0.3	17.9 ± 1.2	< 0.05 (0.0487)[c]	< 0.01 (0.0003)[e]	< 0.01 (0.0028)[f]	-

[a]High glucose DMEM versus low glucose DMEM.
[b]High glucose DMEM versus no glucose DMEM.
[c]High glucose DMEM versus 1 × Locke's.
[d]Low glucose DMEM versus no glucose DMEM.
[e]Low glucose DMEM versus 1 × Locke's.
[f]No glucose DMEM versus 1 × Locke's
