# Supplementary material for: Amino acids serve as an important energy source for adult flukes of Clonorchis sinensis
Source: PLoS Negl Trop Dis. 2020 Apr 30;14(4):e0008287. doi: 10.1371/journal.pntd.0008287 (PMC7217481; doi:10.1371/journal.pntd.0008287)
Supplement: S4 Table — Survival time was evaluated by the log-rank test. P < 0.01 and P < 0.0001 were represented as statistical significance. (PDF) [file pntd.0008287.s014.pdf]

S4 Table. Survival time of *C. sinensis* adults with or without MB05032 treatment.

	Median survival time (days ± SD)	Max survival time (days ± SD)	*P* value
**High glucose DMEM**	20.0 ± 0.6	26.2 ± 2.0	< 0.0001[a]
**High glucose DMEM + MB05032**	7.5 ± 0.2	14.1 ± 1.7	-
**Low glucose DMEM**	25.9 ± 1.0	33.9 ± 3.0	< 0.0001[b]
**Low glucose DMEM + MB05032**	7.0 ± 0.1	12.0 ± 0.7	-
**No glucose DMEM**	23.0 ± 0.5	29.3 ± 1.5	< 0.0001[c]
**No glucose DMEM + MB05032**	5.9 ± 0.1	10.7 ± 0.3	-
**1 × Locke's**	13.0 ± 0.3	17.5 ± 1.5	< 0.01 (0.0011)[d]
**1 × Locke's + MB05032**	3.1 ± 0.1	7.0 ± 0.1	-

[a]High glucose DMEM versus high glucose DMEM + MB05032.

[b]Low glucose DMEM versus low glucose DMEM + MB05032.

[c]No glucose DMEM versus no glucose DMEM + MB05032.

[d]1 × Locke's versus 1 × Locke's + MB05032.
