# Supplementary material for: Amino acids serve as an important energy source for adult flukes of Clonorchis sinensis
Source: PLoS Negl Trop Dis. 2020 Apr 30;14(4):e0008287. doi: 10.1371/journal.pntd.0008287 (PMC7217481; doi:10.1371/journal.pntd.0008287)
Supplement: S1 Fig — (A) A C. sinensis-infected bile sample. (B) An uninfected bile sample. (C) The standard sample. Concentration of free amino acids in bile samples was calculated by using the standard curves generated by the standard sample. (PDF) [file pntd.0008287.s001.pdf]

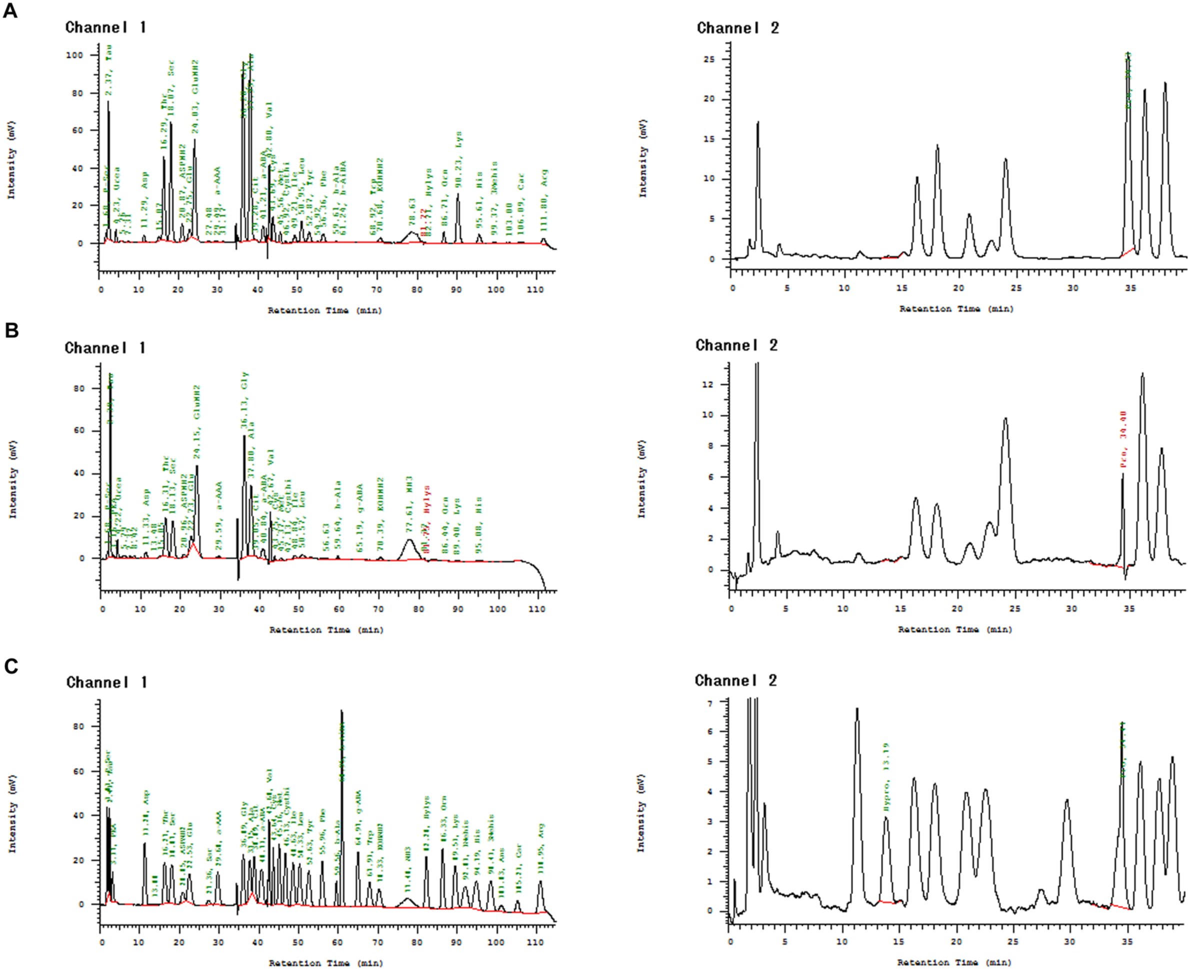
A
Channel 1
Channel 2
B
Channel 1
Channel 2
C
Channel 1
Channel 2
Intensity (mV)
Retention Time (min)
